# Unveiling the Multifaceted Pharmacological Actions of Indole-3-Carbinol and Diindolylmethane: A Comprehensive Review

**DOI:** 10.3390/plants14050827

**Published:** 2025-03-06

**Authors:** Yadava Srikanth, Dontiboina Harikrishna Reddy, Vinjavarapu Lakshmi Anusha, Naresh Dumala, Matte Kasi Viswanadh, Guntupalli Chakravarthi, Buchi N. Nalluri, Ganesh Yadagiri, Kakarla Ramakrishna

**Affiliations:** 1 KL College of Pharmacy, Koneru Lakshmaiah Education Foundation, Vaddeswaram 522302, India; srikanthyadava178@gmail.com (Y.S.); dharikrishana0001@gmail.com (D.H.R.); anushapharmacy90@gmail.com (V.L.A.); nareshpharma2020@kluniversity.in (N.D.); mkasiviswanadh@kluniversity.in (M.K.V.); chakra_varthi123@kluniversity.in (G.C.); buchinalluri@kluniversity.in (B.N.N.); 2 Obstetrics and Gynecology, The Ohio State University Wexner Medical Center, Columbus, OH 43210, USA

**Keywords:** cruciferous vegetables, indole-3-carbinol, diindolylmethane, antioxidant, immunomodulation, organ protection

## Abstract

Cruciferae family vegetables are remarkably high in phytochemicals such as Indole-3-carbinol (I3C) and Diindolylmethane (DIM), which are widely known as nutritional supplements. I3C and DIM have been studied extensively in different types of cancers like breast, prostate, endometrial, colorectal, gallbladder, hepatic, and cervical, as well as cancers in other tissues. In this review, we summarized the protective effects of I3C and DIM against cardiovascular, neurological, reproductive, metabolic, bone, respiratory, liver, and immune diseases, infections, and drug- and radiation-induced toxicities. Experimental evidence suggests that I3C and DIM offer protection due to their antioxidant, anti-inflammatory, antiapoptotic, immunomodulatory, and xenobiotic properties. Apart from the beneficial effects, the present review also discusses the possible toxicities of I3C and DIM that are reported in various preclinical investigations. So far, most of the reports about I3C and DIM protective effects against various diseases are only from preclinical studies; this emphasizes the dire need for large-scale clinical trials on these phytochemicals against human diseases. Further, in-depth research is required to improve the bioavailability of these two phytochemicals to achieve the desirable protective effects. Overall, our review emphasizes that I3C and DIM may become potential drug candidates for combating dreadful human diseases.

## 1. Introduction

Drug discovery and development from natural products has globally gained significant attention against cancers, neurological diseases, cardiovascular diseases, metabolic diseases, infectious diseases, etc. [1,2]. Cruciferous vegetable consumption, such as Brussels sprouts, mustard greens, radish, rutabaga, turnip, cabbage, cauliflower, and broccoli, is reported to have numerous health benefits against these diseases due to their richness in various phytochemicals [3,4]. Among these phytochemicals, indole-3-carbinol (I3C) and 3,3′-diindolylmethane (DIM) have been reported to have multiple health benefits [3] and are currently used as nutritional supplements to prevent hormonal imbalances [5], weight gain [6], and cancer [7,8], and are also used as immunomodulators [9]. The majority of these studies have investigated the anticancer effects of I3C and DIM against various cancers including breast, prostate, and liver, as well as other cancer types [4]. Given the importance of their pharmacokinetic profile and pharmacological actions, I3C and DIM are considered to be potential anticancer agents. Alongside the cancer studies, research about the effect of these two compounds on non-cancerous diseases is simultaneously growing. Currently, few documents report the brief actions of I3C and DIM on non-cancerous diseases [10,11,12]. Indeed, these reports have focused only on a limited number of diseases and have not comprehensively reviewed their protective mechanisms. Across the world, numerous researchers are investigating these two compounds to tackle a variety of diseases, including cancer. Nevertheless, there is a lack of articles in the existing literature that address their sources, biosynthesis, pharmacokinetics, pharmacological applications, and associated toxicities. Consequently, in this thorough review, we have compiled the information related to these two compounds on non-cancerous diseases, which will enable global researchers to understand and apply the current knowledge of these two compounds in their respective research domains. Therefore, in this comprehensive review, we have summarized the current knowledge of the protective effects of I3C and DIM on cardiovascular diseases, neurological diseases, liver diseases, dental issues, metabolic disorders, microbial infections, immune deficiencies, inflammatory diseases, drug-induced toxicities, and radiation-induced complications. Further, in this review, we also concisely explain the toxicities reported so far on I3C and DIM. Overall, this study marks a sublime effort to provide an in-depth review of I3C and DIM and their usefulness in combating various non-cancerous diseases. A comprehensive literature search was conducted to explore the non-anticancer properties of I3C and DIM using databases such as Pubmed, Scopus, Google Scholar, and Web of Science, with keywords including “Indole-3-carbinol” and “Diindolymethane” and those related to their biological activities (anti-inflammatory, neuroprotective, antimicrobial, cardioprotective, antioxidant, etc.), excluding anticancer effects. Studies were included if they were peer-reviewed, published in English, and focused on non-anticancer therapeutic effects in preclinical and clinical models. Exclusion criteria included studies centered on anticancer properties, non-peer-reviewed articles, unrelated biological effects, and low-quality or inconclusive research.

## 2. Biosynthesis and Bioavailability of I3C and DIM

**Figure 1:**
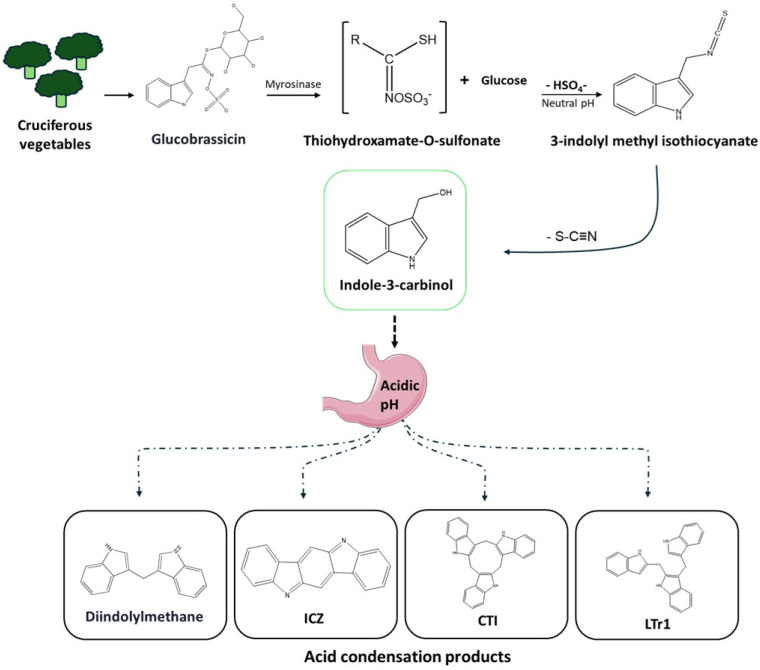
Biosynthesis of indole-3-carbinol and its metabolites. Glucobrassicin from cruciferous vegetables is hydrolyzed by myrosinase, forming thiohydroxamate-O-sulfonate, which decomposes to 3-indolylmethyl isothiocyanate under neutral pH. In acidic conditions (stomach), this converts to I3C, which undergoes condensation reactions. I3C forms diindolylmethane (DIM) and other major acid condensation products like indole(3,2-b) carbazole (ICZ), 2-(Indol-3-ylmethyl)-3,3′-diindolylmethane (CTI), and 3,3′-Diindolylmethane-derived Linear Trimer (LTr1).

Typically, an amount ranging from 20 to 120 mg of I3C is obtained after consumption of various types of cruciferous vegetables [13]. In the acidic environment of the stomach, I3C undergoes dimerization, resulting in a complex mixture of biologically active compounds referred to as acid condensation products such as DIM, indole(3,2-b)carbazole (ICZ), 2-(Indol-3-ylmethyl)-3,3′-diindolylmethane (CTI), and 3,3′-Diindolylmethane-derived Linear Trimer (LTr1), which are freely noticeable after I3C intake (Figure 1) [14,15,16,17]. As noted in Centofanti et al. (2023), the primary oligomeric compound DIM is formed via an acid-catalyzed reaction, and its biological effects have been linked to significant protective mechanisms, notably in cancer prevention and modulation of immune responses [18]. DIM constitutes approximately 10–50% of I3C’s breakdown products, indicating that the average daily intake of I3C from the diet provides between 2 and 24 mg of DIM. Additionally, I3C was not detectable in the tissues of rodents fed I3C, indicating that DIM may be responsible for mediating the physiological effects of dietary I3C [13,19]. However, there is ample evidence to suggest that I3C, in addition to its acid condensation products, is absorbed from the gut and distributed systemically into well-perfused tissues, such as the liver, lungs, brain, kidney, etc. [20,21]. This raises the possibility of some in vivo pharmacological activity of the parent compound as well [22]. Moreover, I3C bioavailability is low (10–35%) and highly variable, whereas DIM is slightly more predictable but has low bioavailability (1–20%). I3C is metabolized primarily through hepatic phase I and II pathways, inducing cytochrome p450 (CYP) 1A2 and CYP3A4 enzymes, while DIM undergoes hepatic oxidation and glucuronidation. The plasma half-life of I3C is short (1–2 h), while DIM has a longer half-life of 4–8 h, and the total area under the curve (AUC) of I3C in rats was found to be 15.85 ± 4.21 (µg/mL × hr), the mean residual time (MRT) was 1.16 hr, and Cmax and Tmax were found to be less than 30 min [22]. Both compounds were reported to be excreted mainly through feces and urine [21,23]. Indeed, human consumption of I3C produced detectable amounts of DIM. Moreover, the AUC (329 to 3376 h × ng/mL), t ½ (3 to 5 h), and Tmax (2 to 3 h) varied between 400 and 1200 mg doses [23], indicating that while using I3C the above doses can be used to achieve chemoprevention. However, I3C and DIM dose and pharmacokinetics need to be defined for non-cancerous diseases with robust clinical investigations.

## 3. The Protective Effects of I3C and DIM Against Various Diseases

### 3.1. Metabolic Disorders

Diabetes and obesity cases are growing at an alarming rate globally; in particular, their prevalence rates are high in low- and middle-income countries [24]. The pathophysiology associated with diabetes and obesity is multifactorial and complex [25]. Medications used to treat these disorders have to be consumed throughout a lifetime which results in numerous side effects and drug resistance. Therefore, current research is focused on seeking new molecules to prevent and treat these disorders. I3C and its metabolite DIM have been extensively studied in preclinical studies for the treatment of diabetes and obesity.

#### 3.1.1. I3C and DIM Regulate Carbohydrate Metabolism

I3C and its metabolite DIM modulated the glucose, insulin, hemoglobin (Hb), and glycated hemoglobin (HbA1C), decreased the conjugated dienes (CDs), thiobarbituric acid reactive substances (TBARSs), and lipid hydroperoxides (LOOHs), and increased the antioxidant enzyme levels and vitamin C and E content in high-fat diet (HFD)-fed mice [26,27]. Additionally, DIM induced glucokinase and glu-6-phosphate dehydrogenase and suppressed glucose 6 phosphatase and fructose-1, 6-bisphosphatase, thereby regulating the carbohydrate metabolism better than I3C. Therefore, this implies that DIM is more potent than I3C in the regulation of carbohydrate metabolism [27].

Chronic progress of diabetes causes severe complications such as diabetes retinopathy, cardiomyopathy, nephropathy, and neuropathy. In recent findings, I3C treatment significantly suppressed the loss of pericytes and vascular retinal leakage in diabetic retinopathy [28]. The activation of protein kinase C (PKC) in diabetic nephropathy disrupts glomerular blood flow and filtration, resulting in albuminuria. Additionally, it promotes the accumulation of extracellular matrix and deposition of collagen, exacerbating renal pathology [29]. Another factor, transforming growth factor beta 1 (TGF-β1), which promotes renal hypertrophy in diabetics. Hence, inhibition of PKC and TGF-β1 would ameliorate diabetic nephropathy. DIM has been shown to inhibit PKC and TGF-β1 signaling in diabetic nephropathy [30].

#### 3.1.2. Anti-Obesity Effects of I3C

Supplementation of I3C and Rutin lowered hepatic retinyl palmitate by 22–52% compared to rats receiving a diet with adequate fat and reduced the fat content and vitamin A concentration in blood plasma by 12% and in the liver by 37% [31]. In another study, I3C at higher doses (500 and 750 mg/kg) lowered the hepatic microsomal cholesterol and cholesterol ester formation significantly, without effecting the serum cholesterol levels, whereas low doses (100, 250 mg/kg) had no effect on hepatic microsomal cholesterol but decreased serum cholesterol without altering the cholesterol ester hydrolase and cholesterol 7-α hydroxylase activity, indicating that it regulates cholesterol levels [32]. I3C also inhibited the Acyl CoA: cholesterol acyltransferase enzyme system which converts free cholesterol to cholesteryl ester [33] and inhibited triglyceride and lipid synthesis, FASN (diacylglycerol acyltransferase 1 and 2; triglycerides), sterol regulatory element binding protein 1 (fatty acid synthesis), and apoB [34], thereby regulating cholesterol homeostasis. Chang et al. observed that I3C treatment decreased acetyl coenzyme A carboxylase mRNA expression, increased peroxisome proliferator-activated receptor gamma (*PPARγ*) expression in epididymal adipose tissue, decreased body weight and fat accumulation, and infiltrated macrophages in the epididymal adipose tissue of obese mice. Further, I3C improves glucose tolerance and lowers serum glucose, triacylglycerol, insulin, and leptin levels [35]. These findings suggest that I3C exerts anti-obesity actions in mice through multiple mechanisms.

#### 3.1.3. I3C and DIM Regulate Adipocyte Differentiation

Adipocyte alteration can result in increased risk and progression of diabetes and obesity [36]. Adipocyte-derived proteins including leptin, adiponectin, visfatin, and omentin possess antidiabetic activity and some proteins are pro-hyperglycemic such as resistin, inflammatory cytokines, and retinol-binding protein 4 (RBP4) [37,38], which are involved in the pathophysiology of obesity and diabetes. Therefore, targeting adipocytes and their derived proteins in combating obesity and diabetes is a promising therapeutic strategy. Incubating adipocytes with I3C hindered glucose uptake both under normal conditions and when stimulated by insulin. Additionally, I3C elevated triglyceride levels and reduced glycogen levels in the liver. Moreover, I3C did not affect levels of high-density lipoproteins (HDLs), total cholesterol, free cholesterol, or esterified cholesterol in the serum or liver. Furthermore, I3C suppressed adipocyte lipogenesis while increasing lipolysis, indicating that I3C modulates adipocyte-mediated fat regulation [39].

Like I3C, DIM also suppressed the expression of *PPARγ*, ccAAT enhancer binding protein α (*C/EBPα*), fatty acid binding protein 4 (*FABP4*), and perilipin, and stimulated the adenosine monophosphate-activated protein kinase α (AMPKα), thereby activating acetyl coA carboxylase to minimize the fat accumulation in adipocytes. Further, DIM activated the aak-1 ortholog of AMPKα catalytic subunits α1 and α2 in *Caenorhabditis elegans* (*C. elegans)* indicating that DIM also impedes adipogenesis through the activation of AMPKα [40]. Chumboatong et al. showed that DIM inhibited inflammatory mediators like monocyte chemoattractant protein 1 (MCP-1), interleukin 6 (IL-6), and tumor necrosis factor α (TNF-α) with a simultaneous increase in the abundance of insulin receptor substrate 1 (IRS-1) pY612 and protein kinase B (Akt)-1/PKB pT308 in adipocyte co-cultured macrophages, indicating that DIM influences the insulin transduction pathway by exerting an anti-inflammatory effect [41]. DIM inhibited the MDI (Methylisobutylxanthine, dexamethasone, and insulin)-induced 3T3-L1 adipogenesis and HFD-induced obesity in mice through decreasing adipogenic proteins such as PPAR-γ and C/EBPα. Additionally, DIM suppressed the G1 phase (early stage of adipogenesis) and induced post-translational degradation of cyclin D1 by inhibiting ubiquitin-specific peptidase 2 (USP2) activities. However, such effects are not exhibited by I3C [6]. DIM, but not I3C, increased adipocyte differentiation through the upregulation of *PPARγ* and *C/EBPα* and enhanced glucose uptake through increasing the expression of glucose transporter type 4 (GLUT-4) in 3T3-L1 adipocytes, which is accompanied by enhanced phosphorylation of Akt, IRS-1, and insulin receptors in the early differentiation of adipocytes [42].

#### 3.1.4. I3C Disrupts Macrophage- and Adipocyte-Induced Lipid Accumulation

Macrophage-derived IL-6 is reported to enhance lipid accumulation in adipocytes [43]. Therefore, the inhibition of macrophage-mediated inflammation could prevent lipid deposition. I3C inhibited intracellular lipid accumulation in hypertrophied adipocytes through the inhibition of IL-6 in macrophage and adipocyte co-cultures and inhibited the infiltration of macrophages, decreased inducible nitric oxide synthase (iNOS), decreased nitrite content, and increased *PPAR-γ* expression [35]. HFD is commonly used to induce and mimic the human type of obesity in experimental animals [44]. HFD downregulates sirtuin 1 (*SIRT1*), *PPAR-α*, and PPAR γ coactivator α1 (*PGC-1α*). I3C treatment ameliorated the HFD-induced weight gain, visceral fat pad weights, and plasma lipid levels by reducing the expression of adipogenic transcription factors such as *PPARγ2,* leptin, and adipocyte protein 2 (*ap2*) in visceral adipocyte tissue. Further, HFD-upregulated TNF-α, interferon β (*IFNβ*), and interleukin 6 are ameliorated by I3C [45]. In another study, I3C has been shown to bind to SIRT1 deacetylase and activate them in the 3T3-L1 cells. I3C did not inhibit adipocyte differentiation in 3T3-L1 cells in SIRT1 knockdown animals. In addition, I3C reduced the mRNA levels of adipogenic genes that encode *C/EBP α*, *PPAR γ2*, fatty acid synthase (*FAS*), and *ap2* in 3T3-L1 cells, suggesting that I3C-mediated inhibition of adipocyte differentiation (adipogenesis) could be due to the activation of *SIRT1* [46]. In continuation to the above work, Choi et al. also found the inhibition of early differentiation of adipocytes through downregulation of adipogenic factors, such as *C/EBPβ, C/EBPδ, PPAR-γ*, and *C/EBPα* with I3C treatment. Additionally, I3C activated *AMPKα*, thereby reducing the triglyceride synthesis [47]. Together, these findings suggest that I3C inhibited adipogenesis through the activation of SIRT1 and AMPKα.

#### 3.1.5. Miscellaneous Effects of I3C and DIM on Lipid Accumulation

Choline and its metabolites are believed to be involved in the formation of atherosclerotic plaque. Trimethylamine-N-oxide (TAMO) causes atherosclerosis by promoting lipid accumulation in macrophages, foam cell formation, and activation of inflammation [48]. I3C treatment against high-choline-induced ApoE−/− mice showed suppression in lipid influx and reduction in atherogenic markers such as trimethylamine (TMA), TMAO, scavenger receptor A (SRA), and scavenger receptor B (SRB1). Further, I3C treatment enhanced gut *verrucomicrobia* in atherosclerosis conditions. 1-methyladenosine-induced RNA methylation plays a crucial role in the pathophysiology of choline-mediated atherosclerosis. I3C inhibited atherosclerosis effects by modulating 1-methyladenosine. Further in vitro studies revealed that I3C mitigated TMAO-mediated lipid deposition and nuclear factor-kappa B (NF-kB)-mediated inflammation. These findings iterate the fact that I3C exhibits antiatherosclerosis effects by reducing lipid accumulation and inflammation [49]. Sleep deprivation impairs cognition by increasing flavin-containing monooxygenase-3 (FMO3) and TMAO levels, which reduce sterol regulatory element-binding protein 2 (SREBP2) expression and brain cholesterol, causing synaptic damage. DIM treatment in sleep-deprived mice inhibited TMAO and thereby upregulated SREBP2 in the brain, resulting in improved synaptic function and memory. These findings suggest that DIM can be used to mitigate the sleep deprivation associated with cognitive dysfunction [50]. Recent findings suggest that I3C treatment also enhanced various gut species, including *Lactococcus*, *Eubacterium*, and *Coprococcus*, while decreasing the abundance of *Eisenbergiella* and *Rikenellaceae*_RC9_gut_group, thereby attenuating the hepatic steatosis, systemic inflammation, and body weight in HFD-fed mice. I3C significantly increased the expression of claudin 4, occludin, and zonula occludens 1 (ZO-1) proteins while also influencing the metabolism of argininosuccinic acid and galactose. Overall, these findings suggest that I3C shows anti-obesity actions by improving gut microbiota [51].

Cumulatively, these findings suggest that I3C and DIM activated several pathways that inhibited adipocyte differentiation and macrophage-induced inflammation. Additionally, both compounds regulated glucose metabolism and mitigated diabetes-associated complications. As of now, both compounds have been extensively studied for their protective roles in metabolic disorders in preclinical studies. Future studies must investigate their protective roles in combating metabolic disorders in clinical settings.

### 3.2. Liver Diseases

The liver plays a crucial role in the elimination of toxic substances and pharmaceutical agents from the body. Many diseases, infections, and chemicals induce liver toxicity which interferes with the protective and metabolic activity of the liver [52]. Many of the drugs are withdrawn from the market due to their potential to induce hepatotoxicity (Triglitazone and Trovafloxacin, etc.) [53]. Therefore, protecting the liver from such types of consequences is essential to retain the normal physiology of the liver as well as the body. The pathophysiology of hepatotoxicity is complex and factors including oxidative stress, inflammation, mitochondrial dysfunction, and immune deficiency, etc., are contributing to liver damage [54]. Preclinically, hepatotoxicity is induced in animals or hepatic cell lines to study these pathological events with the help of acetaldehyde, ethanol, thioacetamide (TAA), carbon tetrachloride (CCl4), and N-nitrosodimethylamine (NDMA) [55]. By any means, the deposition of these chemicals can induce severe liver injury. Several research findings have suggested that I3C and DIM have potential hepatoprotective actions. Therefore, we have summarized the hepatoprotective actions of I3C and DIM.

#### 3.2.1. Hepatoprotective Effects of I3C Against Chemical, Drug, and Alcohol-Induced Liver Toxicity

CCl4 and NDMA are known to induce liver toxicity via decreasing glutathione (GSH) and increasing lipid peroxidation. Pretreatment with I3C has decreased the degree of centrilobular necrosis, restored the serum aspartate aminotransferase (AST) and alanine transaminase (ALT) levels, increased GSH, and decreased lipid peroxidation (LPO) [56,57], as well as elevated the ascorbate levels in the liver [57]. In a recent study, I3C and DIM showed mitigation of CCl4-induced hepatotoxicity by activating nuclear factor erythroid 2-related factor 2 (Nrf2) and heme oxygenase-1 (HO-1) expression and inhibiting NF-kB, TNF-α, and apoptosis [58]. Further, Shen et al. confirmed the hepatoprotective mechanism of I3C via micro Raman spectroscopy [59]. Trabectedin is an anticancer agent with hepatotoxic effects, which has been reported to increase plasma bilirubin, alkaline phosphatase (ALP), AST, and patchy focal necrosis of bile duct epithelial cells [60]. I3C ameliorated the trabectedin-induced hepatic injury markers listed above without affecting its anticancer activity [61]. Acetaldehyde exposure causes activation of hepatic stellate cells (HSCs) resulting in increased AST, lactate dehydrogenase (LDH), malondialdehyde (MDA), hydroxyproline content, and TGF-1β and decreased matrix metalloproteinase 1 (MMP-1)/tissue inhibitor of metalloproteinase 1 (TIMP-1) [62]. I3C ameliorated the acetaldehyde-induced liver toxicity and also promoted extracellular matrix (ECM) degradation [63]. Another study found that pretreatment with I3C inhibited thioacetamide-induced liver and brain injury by reducing the ammonia levels, inhibiting CYP1E1 activity, inflammation, oxidative stress, and mitochondrial dysfunction [64].

Chronic intake of alcohol causes liver injury via enhancing oxidative stress, inflammation, and apoptosis [65]. I3C attenuated ethanol (EtOH)-induced liver injury and hepatocyte apoptosis, but not steatosis via inhibiting lipid peroxidation, hydrogen peroxide content, CYP2E1, and nicotinamide adenine dinucleotide phosphate hydrogenase (NADPH)-oxidase, and showed anti-inflammatory (decrease in IL-1β and neutrophil infiltration) effects and maintenance of mitochondrial complex I, II, and III protein levels and activities. I3C also attenuated EtOH-induced gut leakiness with decreased serum endotoxin levels by preventing EtOH-induced oxidative stress, apoptosis of enterocytes, and alteration of tight junction protein claudin-1 [66,67]. Further, I3C treatment against acute ethanol-induced hepatotoxicity and acetaldehyde-stimulated hepatic stellate cells using precision-cut rat liver slices showed inhibition of CYP2E1 and TIMP-1, increased MMP-1 and alcohol dehydrogenase (ADH), and decreased ALT and AST [68].

#### 3.2.2. I3C and DIM Effects on Inflammatory Liver Diseases

Staphylococcal enterotoxin-B (SEB) causes the release of proinflammatory cytokine and alteration in immune cell activation [69]. Exposure of SEB to D-galactosamine-sensitized mice resulted in an increase in AST, inflammatory cytokines, and massive infiltration of immune cells to the liver, which were ameliorated by treatment with I3C and its metabolite DIM. I3C and DIM triggered the apoptosis in SEB-activated T-cells through caspase-2 but not caspase-3, 8, and 9 and ameliorated the miR-31 expression, resulting in reduced liver injury [70]. Lipopolysaccharide (LPS) is produced by bacteria in the gut and enters the systemic circulation, thereby causing inflammation, including liver damage by the activation of toll-like receptor 4 (TLR4) causing acute and chronic inflammatory diseases [71,72]. DIM protects the liver from LPS-induced toxicity via inhibiting IL-1 receptor-associated kinase 4 (IRAK4) and TLR4 and causes activation of microRNAs like 106a and 20b, which inhibits TLRs involved in the inflammation process [73].

#### 3.2.3. I3C and DIM Effects on Liver Fibrosis, NAFLD, NASH, and MASLD

Pig serum, CCl4, ethanol, and diet with high fat/low protein are known to induce liver fibrosis. During liver fibrosis, several pathological factors are increased (MDA, hydroxy proline, γ-smooth muscle actin (SMA), necrosis, deposition of collagenous fiber, and α-SMA) and decreased (superoxide dismutase (SOD) and MMP-2) [74,75]. I3C treatment reduced liver fibrosis by inducing apoptosis in HSC-T6 liver cells through RIP1K63 de-ubiquitination by upregulating deubiquitinase cylindromatosis (CYLD) and reduced the Bax/bcl-2 ratio [76]. Similarly, DIM treatment suppressed liver fibrosis by inhibiting TGF-β mediated miR-21 signaling, collagen deposition, and fibrotic markers [77]. Nonalcoholic fatty liver disease (NAFLD) is the most common type of liver disease that accounts for more than 32% of liver disease cases and contributes to nonalcoholic steatohepatitis (NASH) [78]. NASH and NAFLD contributed to reducing the gut microbiota, thereby causing lipid accumulation [79]. I3C in combination with chlorogenic acid (CGA) against diet/chemical-induced nonalcoholic steatohepatitis (NASH) showed reduced hepatic lipid accumulation, prevented HSC activation and fibrosis, decreased hepatic CD68-positive macrophages, cleaved caspase -3, and lowered malondialdehyde levels in NASH. Moreover, this combination also restored the relative abundance of gut microbiomes such as *alistipes*, *allobaculum*, *bacteroides*, and *odoribacter* bacteria. These findings suggest that I3C and CGA mitigated NASH by enhancing gut microbiota [80]. Despite hepatoprotection, I3C promoted liver enlargement via an increase in expression of glutamate inotropic receptor n-methyl-D aspartate (NMDA)-type subunit 2C GRIN2C, which encodes NMDA receptor subunit, which is taken into consideration while addressing hepatoprotection [81]. Metabolic dysfunction-associated steatotic liver disease (MASLD) prevalence rates are increasing across the globe. I3C supplementation to HFD-fed mice alleviates liver steatosis, enhances innate lymphoid cells (ILC1 frequency), and activates the AhR. Further, molecular docking analysis showed that I3C binds to the residues of LEU309, TYR316, PHE348, ALA349, and PHE318 on AhR through hydrogen bonds, Π bonds, and hydrophobic bonds, indicating that I3C can be a therapeutic option for MASLD [82].

Accumulation of these results suggests that I3C and DIM show hepatoprotection via modulating oxidative stress, inflammation, mitochondrial dysfunction, and apoptosis and improving gut microbiota, emphasizing that both compounds are potent hepatoprotective agents. Though numerous studies have supported I3C and DIM as hepatoprotective agents based on preclinical investigations, their clinical efficacy in protecting the liver from various toxicants and infections is yet to be studied. Hence, future studies should investigate the clinical potential of I3C and DIM against liver diseases.

### 3.3. Neurological Diseases

Neurological diseases such as Parkinson’s disease (PD), Alzheimer’s disease (AD), stroke, and aging-related diseases are emerging causes of death and disability around the globe [83]. Many of these neurological diseases have no cure except symptomatic relief. Therefore, the identification of potential neuroprotective agents against these neurological complications is highly essential [84]. I3C and DIM have been studied for neuroprotective mechanisms in various neurological diseases [3].

#### 3.3.1. Anti-Ischemic Effects

Vascular damage to the blood vessels that supply blood to the brain causes platelet aggregation and thrombus formation at the site of damage leading to ischemic stroke, resulting in motor, cognitive, and memory dysfunction [3,85]. Thrombolytics, antiplatelets, and antithrombotics are routinely used to treat stroke patients in intensive care units and are recommended to prevent the recurrence of stroke [86,87]. However, these agents have several disadvantages including gastric bleeding and a narrow therapeutic window, particularly for recombinant tissue plasminogen activator (rtPA). Therefore, newer medications with no or minimum adverse effects are sought. I3C has been reported to inhibit platelet aggregation induced by collagen, adenosine diphosphate (ADP), thrombin, and arachidonic acid (AA), thereby preventing thrombus formation [88]. Further, its treatment of middle cerebral artery occlusion (MCAO)-induced experimental stroke rats inhibited platelet aggregation and thrombus formation, which are accompanied by recovery of neurological deficits [22,88].

Indeed, DIM was speculated to mediate the pharmacological actions of I3C [16]. To support this statement, research findings suggest that compared to intravenous administration, oral administration of I3C to MCAO rats significantly escalated the levels of DIM, indicating that DIM may be responsible for I3C’s pharmacological activities [22]. Similarly to its parent compound, DIM also inhibited the platelet aggregation induced by ADP, collagen, thrombin, and AA and ameliorated the formation of thrombus and mitigated neurological deficits in MCAO rats [22]. Rzemieniec et al. found that DIM reduced the ischemia in hippocampal neurons by inhibiting autophagy and apoptosis by mitigating the aryl hydrocarbon receptor (AHR)/CYPIA1 pathway and enhancing histone deacetylase (*HDAC*) activity [89]. I3C treatment against cerebral ischemia/reperfusion injury (CIRI) in rats and mice showed a reduction in inflammatory parameters, oxidative stress markers, and apoptosis intensity. In addition, myeloperoxidase activity, proinflammatory cytokines (IL-1β, IL-6, and iNOS, and elevated anti-inflammatory cytokines such as IL-4 and IL-10), mRNA levels, and NF-kB were also decreased in CIRI rats treated with I3C. Further, caspase activity and apoptosis-inducing factor expression were reduced in treatment with I3C [90,91]. In vitro studies suggest that neuronal exposure to ischemic reperfusion (IR) injury following treatment with I3C showed decreased inflammatory markers such as TNF-α, NF-kB, macrophage-1 antigen (Mac-1), intercellular adhesion molecule-1 (ICAM-1), and lymphocyte function-associated antigen-1 (LFA-1) [91,92]. The hyperactivation of these microglial cells in the brain leads to neuroinflammation, particularly in cerebral ischemia [93]. I3C has been shown to reduce the expression of several apoptotic proteins including Bax, caspase-3, and caspase-9, while increasing the expression of Bcl-2 (antiapoptotic protein). Pretreatment with I3C reduced several inflammatory markers including IL-1β, IL-6, and iNOS and increased the expression of IL-4 and IL-10. The microglia (M1)-induced ionized calcium-binding adapter molecule 1 (IBA1) expression was decreased and M2 microglial marker CD206 expression increased upon pretreatment with I3C, indicating that I3C exhibited neuroprotection through alleviating inflammation and apoptosis [91]. DIM activates the AHR, which effectively alleviates neurological impairments induced by intra-cerebral hemorrhage (ICH) in mice. This activation of AHR not only improves motor function affected by ICH but also inhibits the upregulation of IL-6 and C-X-C motif chemokine ligand 1 (CXCLI), reduces microglial infiltration, and prevents neuronal loss within the hematoma [94]. In addition, both I3C and DIM mitigated oxidative stress, inflammation, and apoptosis in ischemic stroke [88,90,91,95], emphasizing that both I3C and DIM could act as neuroprotective agents. Indeed, as a primary treatment for stroke patients, thrombolytics or antiplatelet, and antithrombotic are recommended, while neuroprotective agents are usually recommended to prevent further brain damage via inhibiting oxidative stress, inflammation, and apoptosis [96]. The above literature confirms that I3C and DIM exhibit antiplatelet, antithrombotic, and neuroprotective mechanisms, emphasizing that both compounds can be used in primary and secondary therapy in stroke.

Hypoxia is the condition where the cells are not able to work efficiently due to a decrease in oxygen supply. Hypoxia following perinatal asphyxia produces significant neurodevelopmental deficits in newborns including motor, cognitive, and other complications. As of now, there is no treatment for perinatal asphyxia and neonatal anoxia, but hypothermia therapy is commonly employed in clinics [97,98]. Therefore, abolishing these neurodevelopmental deficits with the intervention of pharmaceutical agents is an emerging area. I3C oral treatment ameliorated sensory motor neurodevelopment deficits by the mitigation of hypoxia-inducible factor-1α (HIF-1α), mitochondrial dysfunction, oxidative stress, and apoptosis [99]. Like its precursor, DIM downregulated HIF-1α in perinatal asphyxia and inactivated the AHR/NMDA signaling and hypermethylation of specific genes (*AHR* and *GRIN2b*) [100]. Both I3C and DIM distinctly protect adult and neonatal rats by reducing the expression of HIF-1α protein and mRNA. Rzemieniec et al. reported that DIM treatment to mouse embryonic neuronal cells in primary cultures and hippocampal neurons exposed to hypoxic injury showed increased mitochondrial membrane potential (MMP), decreased mRNA expression of AHR and aryl hydrocarbon receptor nuclear translocator (*ARNT*), and mitigated DNA fragmentation [101,102], implying that DIM could impede hypoxic injury in neurons and it can be considered as a potent neuroprotective agent. Together, these findings indicated that I3C and DIM treatment would be advantageous for treating neurological diseases such as cerebral ischemia, neonatal anoxia, and perinatal asphyxia in both adults and neonates. However, robust clinical studies are needed to design and evaluate the clinical efficacy of these compounds against hypoxic injuries and stroke.

#### 3.3.2. Anti-PD Effects

Parkinson’s disease (PD) is characterized by motor and cognitive impairments due to a loss of dopaminergic neurons in the substantia nigra [103,104]. To date, there has been no cure for PD except symptomatic management [104,105]. Therefore, effective PD treatment is required. LPS is commonly used to induce PD in studies aimed at investigating the impact of neuroinflammation on the loss of dopaminergic neurons [106]. Following I3C treatment, LPS-induced PD rats showed decreased oxidative stress and inflammation and showed equipotential improvement in learning behavior, motor coordination, and memory, as seen in levodopa–carbidopa combination therapy [107]. Similarly, I3C treatment in rotenone-induced PD rats shows increased striatal dopamine content with a simultaneous increase in tyrosine hydroxylase (TH) positive cell counts and a decrease in α-synuclein (α-Syn), through stimulation of the SIRT1/AMPK pathway [108]. Metformin is a mitochondrial complex I inhibitor; however, it has been reported that it mitigates the other complications associated with PD [109]. When I3C was simultaneously supplied with metformin, it induced significant improvement in mitochondrial complex I enzyme activity and mitigated the oxidative stress and inflammation in 6-hydroxydopamine (6-OHDA)-induced PD rats, confirming that metformin and I3C combination is a promising therapeutic approach to overcome the metformin-induced mitochondrial complex I toxicity in PD [110]. Indeed, there are no reports on the neuroprotective effects of DIM against PD. Therefore, future studies to investigate the role of DIM in PD are required. However, the para phenyl substituted derivates of DIM mitigated the MPTP-induced inflammation and neurotoxicity in mice [111].

#### 3.3.3. Anti-AD and Dementia

Cognitive and memory impairment are commonly associated with dementia disorders such as Alzheimer’s disease (AD). As of now, there is no treatment available for AD, except symptomatic relief necessitating further need for novel and safe memory-enhancing and anti-AD agents [84]. Morshedi et al. showed that I3C delayed the formation of amyloid beta (Aβ) [112] and halted Aβ1–140 aggregation and its toxicity [113]. Acetylcholine (Ach) is the neurotransmitter that is involved in cognition and memory. Ach levels are regulated by enzymatic activity of acetylcholinesterase (AchE), which degrades the Ach thereby causing cognition and memory impairment. Therefore, the inhibition of AChE enzyme activities is considered a potential target for an improvement in memory [114,115]. In a recent study, I3C showed significant inhibition of AChE and butyrylcholinesterase (BChE) enzyme activities which catalyze the acetylcholine metabolism linked to AD. I3C has also been shown to have inhibitory effects on monoamine oxidase A and B (MAO-A and MAO-B) isoenzymes which play a crucial role in diseases such as PD and AD [116]. Further, it has been reported that I3C and DIM mitigated the AChE and self-induced Aβ aggregation and rescued the SHS5Y5 cells from Aβ-induced neurotoxicity [117]. Further, recent studies show that I3C mitigated cognitive and memory impairment in global cerebral ischemic rats through attenuating AChE activity, oxidative stress, inflammation, and apoptosis [118]. These findings suggest that I3C serves as a neuroprotective agent against neurodegenerative diseases such as PD and AD. However, a thorough investigation of the neuroprotective mechanisms of I3C and DIM is required at preclinical and clinical levels.

#### 3.3.4. Antinociceptive Effects

Nociception occurs as a manifestation of neurodegeneration or inflammation, leading to pain, which impairs quality of life [119]. I3C nanocapsules against formalin-induced pain in rats showed a reduction in pain which was brought by the inhibition of inflammation [120]. Similarly, DIM nanocapsules attenuated the complete Freund adjuvant (CFA)-triggered paw inflammation, relieved formalin-triggered neurogenic and inflammatory pain, and prolonged the pain relief for at least 8 h [121]. Another study found that DIM ethyl cellulose microparticles reduced pain [122]. These findings suggest that both I3C and DIM could alleviate the pain. However, an in-depth understanding of pain management by these two compounds is to be investigated in future studies.

#### 3.3.5. Antidepressant and Anxiolytic Effect

Social isolation is the major risk factor for anxiety and depression [123]. Brain-derived neurotrophic factor (BDNF) is a neurotropic factor that promotes neuronal regeneration [124]. The decline in BDNF levels is associated with depression and anxiety, which highlights the necessity for an enhancement in BDNF [125]. I3C treatment against chronic social defeat stress (CSDS) showed mitigation of depression-like behavior, but not anxiety-like behavior, which are due to an increase in BDNF levels and inhibition of oxidative stress and inflammation in the hippocampus and prefrontal cortex [29]. Similarly to I3C, DIM also enhanced BDNF levels and activated the TrkB/Akt pathway in hippocampal cells, thereby enhancing antioxidant enzymes and mitigating apoptosis [126]. Furthermore, DIM reversed depressive behaviors induced by unpredictable chronic mild stress (UCMS), particularly anhedonia-like behavior, in female mice, without altering anxiety and spatial learning [127]. In a recent study, DIM has been shown to activate the AHR pathway, which is partially protected against serotonergic damage caused by the activation of the kynurenine pathway induced by 3,4-methylenedioxymethamphetamine (MDMA) [128]. These findings suggest that I3C and DIM inhibited stress-induced behavioral changes by enhancing BDNF and inhibiting oxidative stress and inflammation. However, future studies must address the impact of these two compounds on mood disorders.

#### 3.3.6. Miscellaneous Neuroprotective Effects

Excessive glutamate content is considered a pathological inducer of stroke, PD, AD, epilepsy, and other neurological diseases [129,130]. Therefore, inhibition of glutamate action is crucial in acquiring neuroprotective mechanisms. I3C inhibited glutamate-induced excitotoxicity by reducing oxidative stress and apoptosis in PCl2 [131] and HT-22 cells [126]. Clonidine is an antihypertensive agent and its long-term usage is reported to produce significant neurotoxicity via inflammation, oxidative stress, apoptosis, and increased serotonin and noradrenaline levels. I3C treatment significantly mitigated these pathological events [132]. Recent studies have also reported that I3C protected the brain from thioacetamide (TAA)-induced acute hepatic encephalopathy by mitigating hyperammonemia, inflammation, oxidative stress, and apoptosis [64]. Overall, these findings suggest that I3C and DIM inhibited glutamate, clonidine, and TAA-induced neurotoxicity.

Neurodegeneration occurs due to compromised immune responses from microglia [133]. AHR is known for its immunomodulation roles at the central and peripheral levels via regulating inflammation and oxidative stress. Being an agonist of AHR, I3C treatment shows inhibition of retinal degeneration through suppressing microglial activity, inflammation, and oxidative stress via modulation of AHR [134]. Yang et al. showed that DIM treatment reduced the severity of autoimmune encephalomyelitis (EAE) by rebalancing regulatory T cells (Tregs) through indirect inhibition of TH17 cell generation and the production of proinflammatory cytokines which is achieved by activating AHR [135]. BR4044, a novel formulation that was developed to solubilize DIM treatment, reduced brain edema, cortical contusion size, neuronal loss, and microbleeding. It also preserved sensorimotor function and memory. The findings suggest that BR4044 improves neurological outcomes by modulating inflammation and promoting neuronal survival by modulating AHR following traumatic brain injury [136]. Overall, these findings suggest that I3C and DIM inhibited drug- and chemical-induced neurotoxicity and improved the immune function regulating the inflammatory cells in the brain through activating AHR.

In summary, I3C and DIM provide extensive neuroprotection against various neurological diseases including stroke, PD, AD, depression, stress, and drug-induced neurotoxicity. Preclinical studies further indicated that I3C and DIM exert neuroprotective effects by suppressing inflammation, oxidative stress, mitochondrial dysfunction, and apoptosis. Given the importance of these neuroprotective effects, there is considerable opportunity for I3C and DIM to advance from bench to bedside. Hence, the scientific community is encouraged to explore I3C and DIM as viable compounds in the treatment of neurological disorders.

### 3.4. Cardiovascular Diseases

#### 3.4.1. Cardiac Remodeling and Hypertrophy

Cardiovascular diseases are the leading cause of death around the globe and their prevalence rates are increasing day by day [137]. It is well known that cardiac remodeling and altered myocardial metabolism trigger heart failure [138,139] which is accompanied by oxidative stress, cell death, inflammation, imbalanced energy metabolism, calcium transport, contractile proteins, and neuronal activation [140]. Aortic bounding (AB) mimics cardiac remodeling in experimental animals and is thereby used to study cardioprotective effects. Deng et al. identified that I3C-fed AB mice showed increased left ventricular ejection fraction (LVEF) and left ventricular fractional shortening (LVFS) and decreased left ventricular end-diastolic diameter (LVDd), left ventricular end-systolic diameter (LVDs), heart weight/body weight (HW/BW), lung weight/BW (LW/BW), and HW/hindlimb length ratios (HW/HL). Further, I3C attenuated mRNA expression of long-chain acyl coA dehydrogenase (LCAD), CD36, fatty acid binding protein 3 (FABP3), PPAR α, PGC-1α, CTT-1, medium-chain acyl coA dehydrogenase (mCAD), and GLUT-4 [141]. In continuation to the above findings, the same research group identified that knockdown of AMPK-α2 aggravated cardiac remodeling induced by AB. Further, I3C treatment prevented and reversed cardiac remodeling induced by AB through the activation of AMPK-α signaling [142]. Similarly, DIM also enhanced myocardial energy metabolism in a model of pressure overload-induced cardiac remodeling by activating AMPK-α signaling, suggesting that both I3C and DIM activate AMPK-α signaling, thereby effectively preventing cardiac remodeling [141]. Cardiac remodeling leads to hypertrophy, often induced by Angiotensin II (Ang II). Therefore, in experimental settings, Ang II is regularly employed to induce cardiac hypertrophy [143]. I3C and DIM have dose-dependently reduced the angiotensin II (Ang II)-induced increase in atrial natriuretic peptide (ANP), brain natriuretic peptide (BNP), and β-myosin heavy chain (MHC) in H9C2 cells. Further, DIM stimulated p-AMPK-α and disrupted the mammalian target of rapamycin (mTOR) and MAPK signaling response to Ang II, confirms the amelioration of cardiac hypertrophy [144]. Further various studies have reported that DIM activated AMPK-α and inhibited inflammation (TNF-α, IL-6, and NF-kB), oxidative stress, and apoptosis (decreased Bax expression and increased Bcl-2 expression) in H9C2 cardiomyocytes in H9C2 cardiac cells [145]. DIM showed a reduction in TGF-β1-induced conversion of cardiac fibroblasts into myofibroblasts and reduced the mRNA and protein expressions of α-SMA and fibrosis markers like collagen I, collagen III, and connective tissue growth factor (CTGF); it also attenuated the phosphorylation of Akt and glycogen synthase kinase-3β (GSK-3β) and mitigated myoblast differentiation and excessive ECM production through downregulation of Akt/GSK-3β signaling [146]. These reports suggest that I3C and DIM could mitigate inflammation, oxidative stress, and apoptosis in cardiomyocytes, thereby inhibiting cardiac remodeling and hypertrophy. However, further studies need to investigate the therapeutic potential of these two compounds against cardiac remodeling and hypertrophy.

#### 3.4.2. Cardioprotection Against Doxorubicin-Induced Cardiotoxicity

Cancer treatment is usually associated with adverse cardiovascular events which eventually exacerbate the underlying cardiovascular disease [147]. Many anticancer drugs are known to cause cardiotoxicity. Among them, doxorubicin (DOX) is well known to induce cardiac toxicity in human patients and in experimental models by enhancing inflammation and oxidative stress in the heart [148,149]. I3C treatment significantly reduced DOX-induced cardiotoxicity through inhibition of inflammation (IL-6 and TNF-α), oxidative stress (decrease in MDA, increase in catalase (CAT) and SOD), and apoptosis (caspase-3) in mice [150,151]. Moreover, a combination of DOX and I3C treatment showed better anticancer activity than their individual treatment [152]. Akin to these findings in mice, DOX-induced cardiotoxicity and genotoxicity were prevented with DIM [145]. These protective mechanisms of I3C and DIM are due to the inhibition of oxidative stress via activation of the NRF1/HO-1/NADPH quinone oxidoreductase 1 (NQO1) pathway and inhibition of inflammation in cardiac cells, suggesting that I3C could be a potential cardioprotective agent against DOX [152].

#### 3.4.3. I3C as a Malignant Hypertension Inducer

Cytochrome P4501A1 (CYP1A1) is a metabolic enzyme that catalyzes the oxidation of various endogenous lipophilic compounds and xenobiotics. It is not constitutively expressed but can be significantly induced upon exposure to aryl hydrocarbons like I3C [153,154]. The induction of CYP1A1 is mediated by the AHR, a basic helix–loop–helix transcription factor that binds to specific DNA elements in the CYP1A1 promoter [155]. CYP1A1-Ren2 transgenic rats are developed by introducing the mouse *Ren2* renin gene fused with 11.5 kb fragment of the CYP1A1 promoter into a neutral genomic site on the Y chromosome of the Fischer 344 rat [156]. The Ren2 renin gene is not constitutively expressed in these rats; rather, it is produced, mainly in the liver, only when the CYP1A1 promoter is induced by food administration of aryl hydrocarbons like I3C. Malignant hypertension is associated with a rapid rise in blood pressure, pressure diuresis, severe renal vasoconstriction, ischemia, activation of renin–angiotensin–aldosterone system (RAS), microangiopathy, and hemolytic anemia [157]. In preclinical studies, CYP1A1/*Renin-2* (Ren-2) rats are commonly used to induce malignant hypertension because of the expression of the above pathological hallmarks. Being an AHR ligand, several doses of I3C have been used to induce moderate to malignant hypertension in CYP1A1/*Ren-2* rats: 0.3%, [158,159,160,161,162] 0.1%, [163] 0.125%, [164] 0.15%, [165], and 0.3% [166]. Additionally, I3C supplementation elevated plasma prorenin levels, arterial pressure, and moderate hypertensive renal vasculopathy without signs of glomerulosis in CYP1A1/*Ren2* rats [167]. Further, withdrawal of I3C supplementation in young rats presented with an increase in mean arterial pressure [168]. Therefore, these findings suggest that I3C could be a powerful inducer of malignant hypertension in CYP1A1/*Ren2* transgenic rats. However, the induction of hypertension in non-transgenic animals must be elucidated. Oppositely, the recent findings with I3C treatment showed a significant reduction in platelet aggregation, hypertension, normalized heart rhythm, reduced myocardial infarction, oxidative stress, inflammation, apoptosis, and cardiac architectural aberrations against isoproterenol-induced myocardial infarction (MI) in rats [169].

#### 3.4.4. Actions Against Cardiac Inflammation

WW domain-containing ubiquitin E3 ligase 1 (WWP1) overexpression causes cardiomyocyte inflammation in the early stages of MI [170]. WWPI induces cardiomyocyte inflammation by enhancing Krüppel-like factor 1 (KLF15) to catalyze K48-linked polyubiquitination and degradation, resulting in MI and upregulating p65 acetylation and inflammatory signaling of mitogen-activated protein kinase (MAPK) in ischemic myocardium. Recent studies have confirmed that I3C shows stronger interaction with WWP1 than WWP2. Further, DIM shows potent inhibition of WWP1 than I3C, as evidenced by autoubiquitination activity [171]. I3C treatment significantly inhibited the WWPI overexpression and its mediated upregulation of KLF15-ubiquitination, p65 acylation, and MAPK-induced inflammation in MI [172], suggesting that I3C could be a potential cardioprotective agent against MI.

#### 3.4.5. Antiplatelet, Antithrombotic, and Vascular Effects

Vascular injury attracts the platelet and leads to platelet aggregation and thrombosis, respectively. Phytochemicals are reported to reduce the risk of acute coronary disease incidence which is closely associated with thrombosis [86]. I3C and DIM have been reported to have antiplatelet and antithrombotic activities which are evident from the inhibition of platelet aggregation induced by collagen, thrombin, ADP, and AA, as well as thrombin-induced clot formation [88,173,174]. Urokinase is a thrombolytic agent used to dissolve clots. DIM treatment increased the urokinase-type plasminogen activator levels [175]. DIM administration in a 65-year-old patient (one tablet daily for three to four months) resulted in extensive deep venous thrombosis in his right lower extremity and bilateral pulmonary embolism with probable right middle lobe infarction [176]. However, whether the withdrawal f DIM is associated with reduced risk of deep venous thrombosis pulmonary embolism has not been confirmed. Moreover, there is no other evidence of the risk of thrombosis with the supplementation of DIM. Therefore, future studies need to identify the risk of DIM. Contrary to these reports, Ramakrishna et al. found that DIM significantly ameliorated platelet aggregation and thrombosis [22,95,174].

It has been made evident that NF-kB increases leukocyte–endothelial interaction and leukocyte transmigration resulting in inflammation-induced thrombus generation [177,178]. I3C suppressed the expression of inflammatory markers such as E-selectin, ICAM-1, lymphocyte function-associated antigen 1 (LFA-1), and Mac-1, thereby preserving endothelial health [92]. Another study observed that DIM reduced the neointima/media ratio and proliferating cell nuclear antigen (PCNA)-positive cells, without affecting apoptosis of vascular cells and reendothelialization in carotid artery injury. Moreover, DIM suppressed the phenotypic modulation of vascular smooth muscles through the inhibition of platelet-derived growth factor (PDGF)-Rβ, Akt/GSK-3β, extracellular signal-regulated kinase (ERK1/2), and signal transducer and activator transcription (STAT3), suggesting that DIM protected the carotid arteries from external and internal injuries [179]. Further, I3C dose-dependently inhibited PDGF-BB-induced proliferation of vascular smooth muscle cells by arresting the Go/G1 and S phase. I3C also inhibited CD1, CDK4, and CDK6 and reduced the neointima/media ratio and cells positive for proliferating cell nuclear antigen via inhibition of the Akt/GSK3β pathway [180]. LPS activates macrophage tube formation with the association of an increase in vascular endothelial growth factor (VEGF), nitric oxide (NO), IL-6, and MMPs in endothelial cells [181], which was prevented with I3C treatment.

**Figure 2:**
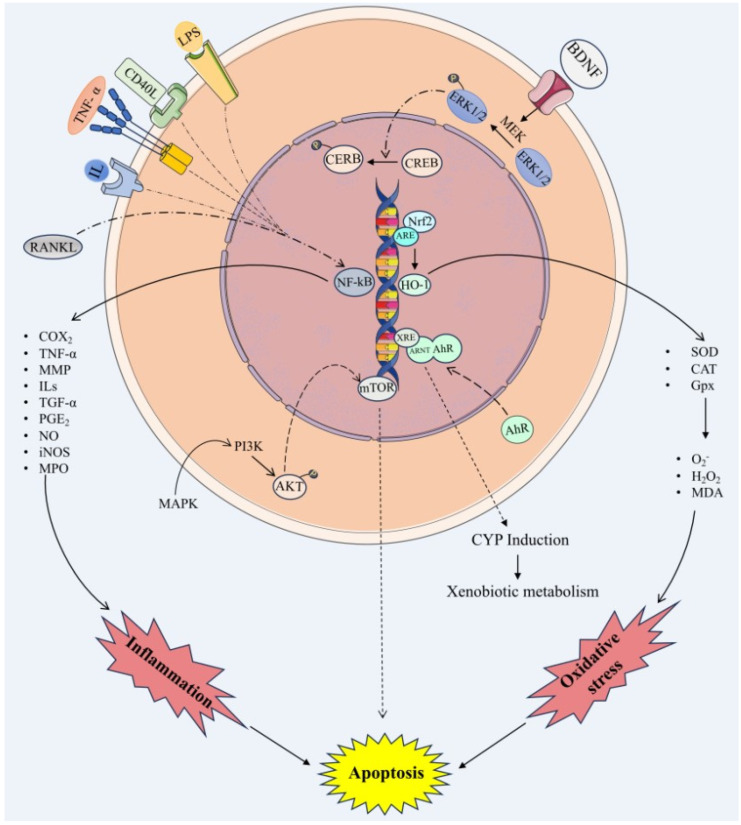
The molecular mechanisms of I3C and DIM. I3C and DIM reduced oxidative stress through the activation of the NRF2/ARE/HO-1 pathway and mitigated the inflammation by blocking various inflammation-inducing factors such as TNF-α, ILs, TLR, RANKL, JAK/STAT pathway, LPS, and CD40, which eventually inhibited the translocation and inhibition of NF-kB. Both compounds activated the BDNF/ERK 1/2/CREBl, MAPK/PI3/AKT/mTOR, and *AHR/ARNT* signaling pathways, thereby controlling cell proliferation, autophagy, apoptosis, and xenobiotic metabolism.

Collectively, these findings suggest that I3C and DIM show antiplatelet and antithrombotic but not thrombolytic activity. Both compounds protect the endothelial cells from inflammatory and oxidative damage, indicating that I3C and DIM can be used to prevent or treat the diseases associated with platelet dysfunction and endothelial dysfunction. Future studies need to be performed to identify the clinical efficacy of these compounds. The molecular mechanisms of I3C and DIM are illustrated in Figure 2.

### 3.5. Microbial Infections

I3C and DIM are reported to halt the growth and invasive nature of various microbial infections including those caused by protozoans, bacteria, fungi, and viruses by interfering with energy metabolisms and replications of these microbial species. The detailed anti-microbial protective mechanisms are discussed below.

#### 3.5.1. Antiprotozoal Effects

Leishmaniasis is a protozoan disease caused by *Leishmania doanvani* [182]. Adenosine triphosphate (ATP) synthase enzyme is essential for the production of energy that leads to Leishmania parasite survival. DIM treatment induced apoptosis in the Leishmania parasite through inhibition of mitochondrial F0F1-ATP synthase [183]. Topoisomerase I maintains the stability and functionality of the Leishmania parasite DNA by promoting DNA relaxation, replication, and transcription processes; therefore, inhibition of this enzyme is a promising therapeutic option to mitigate Leishmania parasite infections [184]. In vitro studies showed that DIM inhibited DNA topoisomerase I in a non-competitive manner resulting in Leishmania parasite death [183]. Another study found that DIM produced random mutations in large and small subunits of Leishmania DNA topoisomerase IB (LdTOP1LS). Leishmania parasites developed these mutations with increased concentrations of DIM as a measure to protect themselves against DIM. Further, it was found that a novel point mutation F270L in the large subunit is responsible for the resistance of the parasite towards the DIM [185], suggesting that Leishmania developed resistance against DIM. Further, Roy et al. developed DIM analogs that have shown antileishmanial activity and are also effective against DIM-resistant Leishmania [186]. These findings suggest that DIM and its analogs show antileishmanial activity. However, further studies need to investigate its beneficial effects in clinics. *Trichomonas vaginalis* is a protozoan parasite that causes trichomoniasis, which is a sexually transmitted disease (STD) [187]. A vaginal Gellan gum-based hydrogel containing I3C-loaded nanocapsules (Eudragit^®^ RS100) and rosehip oil containing I3C reduced the trophozoites’ viability without producing vaginal irritation [188]. Further research is required to identify their clinical potential. *Plasmodium falciparum (P. falciparum)* causes malaria, leading to fatality if untreated [189]. Alothaid et al. (2025) explored the molecular docking and in silico pharmacokinetic properties of I3C and other phytochemicals against the 3D7 strain of *P. falciparum* and various proteins of malaria parasite and demonstrated notable binding, predicting that it can be a potential agent to treat antimalarial candidates [190]. However, future studies need to identify the mechanism of actions of I3C against malaria parasites. Overall, these findings suggest that I3C and DIM can act as antiprotozoal agents.

#### 3.5.2. Antifungal Effects

*Candida albicans* (*C. albicans*) is a type of fungus that is present in small amounts in the human body (mouth, skin, and intestine). Overgrowth of *C. albicans* leads to candidiasis, a fungal infection [191]. I3C exhibited candidacidal activity in an energy-independent manner without causing hemolysis in human erythrocytes. This study further confirmed that the anticandidal activity of I3C could be due to binding to and disruption of DNA [192]. The National Committee for Clinical Laboratory Standard—NCCLS—broth microdilution assay is routinely used to identify the minimum inhibitory concentration of antimicrobial agents [193]. When tested for I3C potential to impede the growth of *C. albicans* in the broth, I3C arrested the cell cycle at the G2/M phase and disrupted the cell membrane structure [194]. These findings suggest that I3C acts as an antifungal agent, particularly against *C. albicans*. Further preclinical and clinical studies need to investigate the complete therapeutic potential of I3C as a candidacidal agent.

#### 3.5.3. Antibacterial Effects

Bacterial infections are very common across the globe. At present, several antibiotics are used as bacteriostatic or bactericidal agents to overcome bacterial infections. Due to the multidrug resistance developed by various bacteria, treating bacterial infections is becoming a global challenge [195]. Therefore, there is an imminent requirement for novel antibacterial agents. Indole and its derivatives show potent antibacterial effects [196]. Sung et al. reported that I3C shows better antibacterial activity against Gram-positive bacteria than Gram-negative bacteria which could be due to the prevention of LPS by I3C. Further, I3C, when combined with ampicillin, synergistically acted against drug resistance, indicating that I3C possesses broad-spectrum antibacterial activity [197]. *Clostridium difficile* (*C. difficile*) is a Gram-positive bacterium that causes colon infection and alters the gut microbiota in recipients following antibiotic therapy [198]. I3C treatment for *C. difficile* infection (CDI) in mice showed decreased mortality and CDI severity and enhanced cecal Tregs, γδ T cells, ILC3s, and neutrophilic response without elevating inflammatory response and bacterial translocation, indicating that I3C has the potential to prevent CDI and improve the gut microbiota [199]. Another study found that ethyl acetate extracts of I3C-exposed *Daldinia eschscholzii* (*D. eschscholzii*) fungal cultures produced two rare and novel indole alkaloids such as dalesindoloids A and B. Both alkaloids showed *Staphylococus aureus* (*S. aureus*) growth inhibition [200]. Similarly, I3C exposure to *D. eschscholzii* fungal cultures also expressed the polyketide-indole hybrids (PIHs), named indolchromins A and B, that were shown to have antibacterial effects as they inhibited *C. difficile*, *C. perfringens*, *Bacteroides fragilis*, *Streptococcus pyogenes*, and *Veillonella* species [201]. These findings suggest that I3C can be used to produce various antibacterial agents from *D. eschscholzii*. *Escherichia coli* (*E. coli*) is abundantly present in the gut without causing any harmful effects. However, their overgrowth and some of the serotypes of *E. coli* that are contaminated through food or water cause diarrhea or food poisoning [202]. Therefore, the inhibition of *E. coli* and its variants would prevent food poisoning. I3C inhibited *E. coli* growth through inducing DNA damage and reactive oxygen species (ROS)-mediated cell death by enhancing hydroxy radicals rather than oxygen radicals [203].

Bacterial species from the biofilm on the surfaces of the host protect them from the host defense system. Therefore, the inhibition of biofilm formation is an important factor for antibiotic agents. Acne vulgaris is a common type of cutaneous inflammatory disorder that occurs because of *Cutibacterium acnes* (*C. acnes*) (an anaerobic Gram-positive bacteria). Biofilms of *C. acnes*, *S. aureus*, and *Candida albicans* (*C. albicans*) are difficult to remove from the skin [204]. I3C and DIM inhibited bacterial growth and biofilm formation of these bacteria. However, DIM was more effective in exhibiting the antibacterial activity and inhibition of biofilm formation by *C. acnes* or its combination with *S. aureus* and *C. albicans*. Further, it was reported that DIM inhibited hyaluronate lyase, virulence-related genes, and lipase that promotes biofilm formation and inhibited the hyphal formation and cell aggregation of *C. acnes* [205]. These findings suggest that I3C and DIM not only show antibacterial activities but also halt biofilm formation. Moreover, DIM has also been shown to inhibit the biofilm formation caused by Gram-negative bacteria such as *Pseudomonas aeruginosa* (*P. aeruginosa*) (70% inhibition) and *Acinetobacter baumannii* (*A. baumannii*) (65% inhibition) and the percentage of inhibition escalated to 94% when DIM was administered with Tobramycin. Further, DIM formulation reduced the *P. aeruginosa*-infected wound size and bacterial burden [206]. Multidrug resistance (MDR) by the use of a combination of multiple antibiotics is a global medical threat nowadays [207]. To overcome MDR, a combination of carbonyl cyanide 3-chlorophenylhydrazone (CCCP) and ethylenediaminetetraacetic acid (EDTA) with I3C showed potent bactericidal activity against MDR Gram-negative bacteria such as *A. baumannii*, *Klebsiella pneumoniae*, *P. aeruginosa*, and *E. coli* compared to being administered alone. Further, this study also suggests that I3C combination with EDTA was more effective in mitigating the MDR [208]. Collectively, these findings suggest that I3C and DIM show antibacterial effects and overcome MDR and biofilm formation. Future studies need to investigate the clinical potential of I3C and DIM in combination with other antibiotics to overcome MDR.

#### 3.5.4. Antiviral Effects

*Human papillomavirus* (HPV) causes recurrent respiratory papillomatosis (RRP) in humans which is characterized by stridor, dyspnea, chronic cough, and recurrent pneumonia [209]. A phase I trial of I3C treatment against RRP shows that 33% (6 out of 18) of RRP patients have a complete cessation of papilloma growth. The protective effects of I3C could be due to changes in the ratio of urinary 2-hydroxylation and 16-hydroxylation of estradiol. These findings showed that I3C may be used to treat RRP but it is recommended to evaluate this using long-term studies [210]. Later, the same research group identified that I3C can be used to treat the RRP without any side effects [9]. I3C treatment significantly mitigated the Herpes simplex virus (HSV) infection strains such as HSV1, HSV2, and HSV1 strains to acyclovir by promoting p53, p21, and cyclin D3, while reducing cyclin A levels, thereby arresting the G1/S phase and eventually inhibiting DNA and the HSV replication cycle [211]. Another study found that DIM oral treatment promoted the clearance of reovirus from the GI tract and elevated the mucosal IgA response [212]. Recent studies showed that I3C treatment of human Lung Organoids (hLORGs) and VeroE6 cell lines (kidney cells) incubated with SARS-CoV-2 (Omicron variant) infection exhibited decreased replication of SARS-CoV-2 which is due to the downregulation of mRNA expression of interferon (IFN)-related genes (interferon-induced protein with tetratricopeptide repeats 1 (*IFIT1*), tripartite motif containing protein 22 (*TRIM22*), and myxovirus resistance protein 2 (*MX2*)) and upregulation of proinflammatory chemokines and cytokine TNF-α, CXCL10, IL-6, and hLORGs, indicating that I3C can inhibit the replication of SARS-CoV-2 [213]. In continuation to the above findings, it was identified that I3C inhibited the NEDD4 E3 ubiquitin protein ligase (NEDD4) and WWP1 enzymes which are essential for viral production and promoted the ubiquitination of virus matrix protein and viral egression [214]. The SARS-CoV-2 main protease (Mpro), a crucial enzyme for viral transcription and replication, is highly conserved across different variants and distinct from human proteases, making it a promising target for therapeutic intervention against SARS-CoV-2. DIM derivatives significantly inhibited the Mpro enzyme, thereby impeding viral transcription and replication [215]. Cumulatively, these findings suggest that I3C produced antiviral effects against HPV, HSV, reovirus, and SARS-CoV-2. Further research is required to identify the molecular mechanisms of action of I3C. The detailed antimicrobial actions of I3C and DIM and their derivatives are depicted in Table 1.

### 3.6. Radioprotection

Radiation therapy is commonly used to remove tumors in cancer patients by causing DNA damage, leading to the destruction of cancer cells as well as the potential damage to healthy cells [216]. Therefore, it is recommended to use a radioprotective agent to overcome the disruptions of healthy cells caused by radiation therapy. DIM activates the phosphorylation of the ataxia-telangiectasia mutated (*ATM*) gene and its subsequent activation of NF-kB signaling and DNA damage response (DDR) in radiation-damaged cells, leading to the stimulation of NF-kB-mediated inhibition of protein phosphatase 2A, an ATM regulator, and repair radiation-induced DNA double-strand breaks (DSBs). Additionally, in non-tumorigenic epithelial cells, DIM increases survival and DSB repair which is dependent on intact *ATM* function, indicating that DIM can act as a radioprotective agent through promoting *ATM*-driven DDR and NF-kB survival signaling [217]. Another study finds that DIM protects the bone marrow cells from total body irradiation (TBI) injury by decreasing oxidative stress (upregulation of NRF2, HO-1, and downregulation of NADPH oxidase 4) and apoptosis (increases antiapoptotic protein Bcl-2 and decreases pro-apoptotic protein Bax) in hematopoietic stem cells, suggesting that DIM could be used as a radioprotectant to alleviate TBI-induced hemopoietic injury [218]. In a study aimed to evaluate the effect of the combination of resveratrol (RES) and DIM on radiation-induced injury, the combination of RES and DIM significantly increased antioxidant enzymes like catalase, SOD, and GPx and also decreased the chromosomal aberrations, as well as micronuclei formation, indicating that a combination of RES and DIM can be used to treat radiation-induced injury [219]. Lu et al. observed that DIM protects intestine cells from ionizing radiation by enhancing Lgr5+ ISCs and their progeny cells, lysozyme+ Paneth cells, Villin+ enterocytes, and Ki67+ instantaneous amplifying cells and increases NRF2 levels, reducing DNA damage, and ROS production, thereby protecting human intestinal epithelial cell-6 (HIEC-6) [220]. The detailed radioprotective mechanism of DIM is reviewed in a report with its pharmacological actions [221].

Cumulatively, these studies confirm that DIM offers prevention and mitigation of tissue damage due to radiation therapy during cancer treatment. Whether I3C produces a similar kind of radioprotection has not yet been studied. Therefore, future studies need to investigate the radioprotective mechanisms of I3C.

### 3.7. Inflammatory and Gastrointestinal Diseases

#### 3.7.1. Anti-Inflammatory Effects Against LPS-Induced Inflammation

Inflammatory mediators such as cytokines, polypeptides, and leukotrienes aggravate the pathophysiological events in many diseases; therefore, targeting these molecules to inhibit inflammation has gained attention in recent years [222]. LPS is a component of the outer membrane of Gram-negative bacteria and protects the bacteria from its surroundings [223]; its administration is reported to activate inflammatory cascades. Therefore, LPS is considered to be an inflammation inducer and can be used to screen the anti-inflammatory activity of new compounds [224]. In a study to assess the anti-inflammatory effect of I3C, it was found that I3C inhibited NO production and also increased the clearance of NO and decreased TNF-α and IL-10 in RAW 264.7 macrophages incubated with LPS [225]. Macrophages contain several structural components that are responsible for immune activity by inducing various inflammatory mediators, thereby eliminating foreign bodies [226]. However, hyperactivation of macrophages induces inflammation, resulting in various inflammatory diseases [227]. I3C attenuated the production of LPS-induced proinflammatory mediators such as NO, IL-6, TNF-α, and IL-1β and suppressed immune cell infiltration in Raw264.7 cells (macrophages) and lungs in broncho-alveolar lavage fluid (BALF) in an LPS-induced acute lung injury mouse model. The I3C-mediated anti-inflammatory effect occurs via regulation of the Toll/IL-1R-domain-containing adaptor-inducing IFN-β (TRIF)-dependent signaling pathway [228].

Like I3C, DIM also decreased NO, prostaglandin E2 (PGE2), TNF-α, IL-6, IL-1β, iNOS levels, and mRNA levels of phospholipase A1 (PLA2), NF-kB, AP-1, and AP-1 DNA binding activity and phosphorylation of c-Jun in Raw264.7 cells treated with LPS, indicating that DIM shows anti-inflammatory activity, which is due to downregulation of NF-kB and AP-1 signaling [229]; it also ameliorated 12-O-tetradecanoylphorbol-13-acetate (TPA)-induced inflammation by reducing cyclooxygenase (COX-2), iNOS, CXCL15, IL-6, and nuclear translocation of p65, ERK ½, and inhibitor of NF-kB (IKB) kinase [230]. It is a well-known fact that microglial hyperactivation causes neuroinflammation, which contributes to neurodegenerative diseases [231]. In vitro studies on BV-2 microglia cells have reported that DIM suppressed the LPS-induced elevation of iNOS and COX-2, thereby protecting the primary cortical neurons and hippocampus from inflammatory injury. DIM, but not I3C, attenuated NF-kB-initiated hyperactivation and neuronal death, suggesting that DIM could be a potent anti-inflammatory agent against LPS-induced neuroinflammation [232]. In contrast, significant inhibition of LPS-induced neuroinflammation was observed in PD with I3C treatment [107]. Cumulatively, these findings suggest that I3C and DIM can be considered as potent anti-inflammatory agents.

#### 3.7.2. Anti-Arthritis Effects

Arthritis is an inflammatory disease and several inflammatory markers are involved in its pathophysiology [233]. Methotrexate (MTX) is the first-line drug for treating rheumatoid arthritis (RA). However, it is associated with hepatotoxicity [234]. I3C with the combination of MTX was shown to reduce hepatotoxicity through the inhibition of inflammation and oxidative stress against complete Freund’s Adjuvant (CFA)-induced arthritis in rats [235]. However, whether I3C mitigated the MTX-induced hepatotoxicity in cancer without altering MTX anticancer efficiency is yet to be investigated. Similarly, DIM treatment to adjuvant-induced arthritis (AIA) and endotoxin-induced bone resorption (EIBR) showed a reduction in inflammatory cytokines, clinical and histologic indices of inflammation, and tissue damage in arthritis through the inhibition of the receptor activator for NF-kB ligand (RANKL) [236]. Another research study has shown that DIM suppressed the proliferation, migration, and invasion of rheumatoid arthritis (RA) fibroblast-like synoviocytes (FLSs) and MAPK/AKT/mTOR pathways, thereby preventing knee joint inflammation [237]. Collectively, this evidence suggests that I3C and DIM can mitigate RA through the inhibition of inflammation. Future studies need to investigate the therapeutic potential of these compounds in clinics.

#### 3.7.3. Inflammatory Skin Diseases

Skin is one of the largest protective organs in the human body. Several toxins, infections, and environmental pollutants are continuous threats to the skin, particularly cutaneous inflammation [238]. Atopic dermatitis is a persistent inflammatory skin disease with multifaceted pathophysiology [239]. In NC/Nga mice stimulated with the 2,4-dinitrochlorobenzen (DNCB) model of atopic dermatitis, oral administration of I3C effectively reduced atopic dermatitis-like skin inflammation by declining serum IgE levels, epidermal thickening, inflammatory cell infiltration, trans-epidermal water loss, and scratching behavior, and decreased proinflammatory cytokine expression, thymic stromal lymphopoietin (TSLP), and periostin against TNF-α- and IFN-γ-induced inflammation in HaCaT keratinocytes. The effects of I3C are attributed to its capability to inhibit the MAPK and NF-kB pathway [240]. Locust bean gum-based hydrogel containing I3C (Eudragit^®^ RS 100) nanocapsules show enhanced anti-inflammatory activity against croton oil and UVB radiation-induced skin damage. Further, these nanocapsule forms of I3C showed a better reduction in edema (86.13 ± 8.43%) and leukocyte infiltration than their nonencapsulated counterparts without skin irritation, suggesting that I3C and its nanocapsule formulations can be used safely to mitigate cutaneous inflammation [241]. Similarly, Vargas et al. observed that I3C in a nanocapsulated hydrogel formulation improved wound healing by lowering the levels of inflammatory markers such as IL-1β by 80.07 ± 9.19% and reduced oxidative damage by lowering ROS, lipid peroxidation, and protein carbonylation with a concomitant rise in catalase activity and vitamin C levels [242]. Similarly, DIM also enhanced wound healing caused by second-degree burns by activating the Wnt/β-catenin (increasing Wnt11)-mediated exosome autocrine signaling pathway [243]. Cumulatively, these findings suggest that I3C and its hydrogel nanocapsule formulations and DIM mitigated skin inflammatory disorders.

#### 3.7.4. Miscellaneous Inflammatory Diseases

Periodontitis is an inflammatory disorder that leads to alveolar bone resorption [244]. DIM shows protection against periodontitis by decreasing the infiltration of inflammatory cells, reducing IL-1β, IL-6, RANKL, and NF-kB levels, retaining connective tissue in the papilla, reducing interproximal and interradicular bone resorption, and inhibiting the LPS-induced elevation of cytokines in human periodontal ligament cells [67,245]. While DIM demonstrates protective effects against periodontitis, further research is needed to fully comprehend its molecular mechanism in this regard.

Inflammation and oxidative stress are associated with polycystic ovarian syndrome (PCOS) [246]. I3C and linagliptin combination increased the SOD, CAT, Nrf2, and HO-1, decreased TGF-β, TNF-α, and IL-10, and reduced the plasma-free testosterone, luteinizing hormone, progesterone, and estradiol in PCOS, suggesting that I3C with linagliptin could be useful to treat PCOS by inhibiting inflammation and accelerating antioxidant levels [247].

Cumulatively, these observations suggest that I3C and DIM are effective against various inflammatory diseases including arthritis, LPS-induced inflammation, inflammatory skin diseases, periodontitis, and PCOS. Moreover, I3C and DIM mitigated inflammation and their associated complications in microbial infections, neurological, cardiovascular, liver, gastrointestinal, dental, bone, and skin diseases, indicating that both compounds can be considered as potent anti-inflammatory agents.

### 3.8. Gastrointestinal Diseases

#### 3.8.1. Anti-Colitis Effects

Acute colitis is an inflammatory disease that involves multiple pathological events, including genetic predisposition, gut dysbiosis, imbalance of the immune system, and deficits in intestinal epithelial barriers which impose a risk of cancer [248]. Therefore, inhibition of colitis is essential to keep the gut environment healthy and prevent the risk of cancer. 2,4,6 trinitrobenzene sulfonic acid (TNBS) is commonly used to induce acute colitis in experimental animals [249]. Being an anti-inflammatory compound, when I3C was tested against TNBS-induced colitis, it reduced the increased body weight and body condition, reduced dehydration, and improved stool consistency in female mice but not in male mice. I3C did not alter TNF-α and IL-12 in male and female mice but increased the IL-1β and IL-6 in male mice and decreased IFN-γ in female and male mice, suggesting that I3C ameliorates colitis in females but not in males, indicating that I3C sex-specifically exhibits anti-colitis activity in mice [250]. However, future studies need to investigate the reason for not exhibiting protective responses in males. Similarly, Palrasu et al. reported that I3C treatment in mice with anti-CD40 or dextran sulfate sodium (DSS)-induced colitis showed a significant increase in mRNA expression of AhR, antimicrobial peptides (AMPs), b-defensins such as Reg4, mBDs, and mucins, indicating that I3C attenuated colitis through the activation of AhR, restoring gut microbiota balance, and preventing inflammation [251]. In support of these findings, a recent study revealed that knocking out the AhR in intestinal epithelial cells in mice and feeding with an AhR ligand-depleted diet showed signs of intestinal inflammation, colitis, and increased levels of LPS in response to chronic DSS, which resulted in increased mortality of IEC-specific AhR knockout (AhRΔIEC) mice. I3C supplementation was shown to reverse these signs and reduce mortality by ameliorating intestinal inflammation and restoring altered gut microbiota, including families of *clostridicae* and *lachnospriaceae*. I3C supplementation to SAMP/YitFc mice with spontaneous ileitis showed significant recovery in epithelial abnormalities, suggesting that I3C mitigated the colitis and gut dysbiosis through the activation of AhR [252].

One study aiming to identify the link between intestinal epithelial cells (IECs) and AHR in mitigating colitis showed that I3C enhanced mucin proteins, thereby promoting goblet cell development in wild-type mice that have colitis. Further, it was confirmed that AhRΔIEC mice showed impaired mucin protein expression, particularly mucin 2 (Muc2), which is independent of IL-22. These findings demonstrate that AhR regulates Muc2 expression, thereby promoting goblet cell development in colitis [253]. Quinolinic acid (QA), a kynurenine pathway metabolite, was found to be upregulated in colitis and linked with the occurrence of depressive-like symptoms. I3C treatment reduced the QA content in mice, reduced the immobility time, and modulated the inflammation through immune cell-mediated interleukin-22 production, suggesting that I3C mitigated depressive-like behavior in colitis [254]. Like I3C, DIM attenuated the DSS-induced colitis by inhibiting TNF-α, IL-6, IFN-γ, IL-10, iNOS, COX-2, NO, PGE2, myeloperoxidase activity (MPO), and NF-kB. DIM attenuated the loss of body weight, shortening of the colon, and clinical signs [255] and also suppressed neutrophil infiltration, VEGF-A, VEGFR-2, VEGF-C, VEGF-D, VEGFR-3, and angiopoietin-2, indicating the inhibition of angiogenesis and lymphangiogenesis [256]. Cumulatively, it was identified that I3C attenuated acute colitis through activating AhR, improving gut microbiota and mitigating inflammation, whereas DIM inhibited inflammation and angiogenesis, revealing that both I3C and DIM can be considered as a potential agent against colitis. Future studies need to investigate the clinical efficacy of these compounds in combating colitis.

#### 3.8.2. Anti-Ulcer and Antidiarrheal Effects

Aspirin is a nonsteroidal anti-inflammatory drug (NSAID) known to induce gastric ulcers. I3C alone or in combination with omeprazole inhibited the aspirin-induced ulcers without any side effects, indicating that I3C can be used as an antiulcer agent [257,258]. Lin et al. (2025) revealed that microbiota-derived I3C plays a key role in ameliorating bile acid disorder-induced diarrhea by regulating gut epithelial glucuronidation. I3C, produced by *Lactobacillus reuteri*, inhibits UDP glucuronosyltransferase 1A4 (UGT1A4), reducing toxic chenodeoxycholic acid-3β-glucuronide levels and activating the Farnesoid X Receptor-Sirtuin 1-Liver Kinase B1 (FXR-SIRT1-LKB1) pathway. This activation enhances gut barrier function and reduces epithelial apoptosis through P53 regulation. These findings highlight I3C’s potential in restoring bile acid balance and gut health [259]. These findings also suggest that I3C can be used to mitigate gastrointestinal diseases such as ulcers, ulcerative colitis, and diarrhea. However, future studies need to address its protective roles in clinical settings.

### 3.9. Immune Modulation

The immune system continuously combats against foreign bodies, thereby protecting the body from harmful substances. A compromised immune system may lead to defensive failure in the body, causing various diseases [260]. Therefore, immunomodulation with external agents would be considered promising for combating various human diseases. I3C is reported to have immunomodulation properties. For example, an I3C oral dose of 150mg/kg for seven weeks reduced natural killer cell-induced cytotoxicity and increased T-cell-mediated delayed-type hypersensitivity with no alteration in antibody production in response to antigen keyhole limpet hemocyanin [261]. Therefore, this study recommends exercising caution in experiments involving immune activities to prevent long-term modifications to the immune system resulting from prolonged interventions with I3C. AhR agonists have an effect on oral tolerance as well as food allergies. I3C increased the expression of the aldh1 gene which led to the formation of retinoic acid, thereby inducing regulatory T cells. I3C lowered the serum ovalbumin IgG1 response in an experiment of oral tolerance and attenuated the symptoms of peanut allergy. Therefore, this study supports the idea that I3C could be an immunomodulator [262].

The spleen is one of the major immune organs that fights against foreign bodies [263]. It was reported that DIM induced the proliferation of splenocytes and augmented mitogen- and IL-2-induced splenocyte proliferation. Further, DIM stimulated IFN-γ production and its receptor expression and stimulated the production of ROS in murine peritoneal macrophage cultures. Furthermore, oral but not intraperitoneal administration of DIM induced elevation of IL-6 by 8-10-fold (peak at 5 h), G-colony stimulating factor (G-CSF) by 10-fold (peak at 3h), IL-12 by 3-fold (sustained for 24 h), and IFN-γ by 2-fold (peak at 24 h) [212], suggesting that DIM could be a potent immunomodulator. LPS is used to induce acute respiratory distress syndrome (ARDS) in experimental animals [264]. I3C mitigated LPS-induced ARDS by decreasing various immune cells such as CD4+ RORγt +IL-17a+IL-22+ pathogenic Th17 cells but not CD4+RORγt +IL-17a+IL-22− homeostatic Th 17 cells in the lungs. I3C increased Th22 cells by 3-fold and IL-22 mRNA by 2.5-fold, enhancing a protective immune response. At the same time, it reduced miR-29b-2-5p by 2-fold and RAR-related orphan receptor C (RORc) by 1.8-fold, suppressing pathogenic Th17 activity and shifting the immune balance toward lung protection. These protective effects of I3C could be due to AhR activation [265]. These findings suggest that I3C regulates lung Th17 and Th22 cells through the activation of AhR in ARDS. In ARDS, CCR2+ monocytes play a role in the recruitment and migration of CXCR2+ neutrophils. Later, the same research group found that I3C decreased the CXCR2-expressing neutrophils and reduced the CCL2 (MCP-1) protein levels of broncho-alveolar lavage fluid in LPS-induced ARDS, possibly due to AhR activation that regulates immune cells transferring to the lung in ARDS [266]. DIM attenuated the vascular leak and IFN-γ secretion was caused by SEB in the lungs through the promotion of miRNA apoptosis and cell cycle arrest in SEB-activated T cells [267], suggesting that DIM can impede bacterial-mediated lung injury by maintaining immune function. Overall, these findings recommend that I3C and DIM may act as immunomodulators.

Systemic lupus erythematosus (SLE) is an autoimmune disease where B cells become overactive and produce excessive amounts of antibodies, leading to systemic failure [268]. I3C treatment in SLE has increased immature B cells and T cells but decreased mature B cells, autoantibodies, mature memory T cells, and the CD4: CDT cell ratio, produced Th1 cytokines, and transiently blocked B cell maturation [269], indicating that I3C can act as an immunosuppressant, mitigating immune cell overactivation in SLE. Estrogen metabolite 16α-hydroxyestrone increases the disease progression of SLE. In female (NZB × NZW) F1 mice that are prone to SLE, I3C treatment changed the ratio of urinary 2:16α-hydroxyestrone levels, indicating that it attenuated the SLE due to its antiestrogenic activity [5]. Both of these findings suggest that I3C could mitigate the SLE through its immunomodulation activities and antiestrogen activities. However, future studies need to fully establish the protective roles of I3C in SLE.

### 3.10. Drug-Induced Toxicities

Several anticancer drugs are reported to have severe cardiotoxic (doxorubicin), nephrotoxic (cisplatin), and hepatotoxic (methotrexate) effects [270]. Because of these side effects, there is a raised concern about patient safety, leading to discontinuation or limiting their use of chemotherapy. Therefore, mitigating their side effects without altering their anticancer properties is of the utmost importance. In this section, we have summarized the I3C and DIM protective mechanisms against various anticancer agents and associated drug-induced toxicities.

Cisplatin is used to treat several cancers including lung, brain, ovarian, breast, biliary, and prostate cancers with significant nephrotoxicity [271]. Cisplatin causes renal epithelial cell damage by inducing oxidative stress, mitochondria, and DNA damage, which eventually leads to renal apoptosis and necrosis [272]. El-Naga et al. reported that cisplatin (7 mg/kg body weight)-induced increases in kidney weight, serum creatinine, and caspase-3 levels are reduced by I3C; antioxidant enzymes such as GSH and SOD are elevated with a simultaneous decrease in lipid peroxidation products and NADPH oxidase 1 (NOX-1) and increase in epidermal growth factor (EGF), insulin growth factor 1 (IGF-1), IGF-1R, and calcitonin gene-related peptide (CGRP) [273], suggesting that I3C ameliorated the cisplatin-induced acute nephrotoxicity. It is noteworthy to mention that the coadministration of cisplatin with I3C inhibited the tumor cell growth in prostate cancer with diminished nephrotoxicity without altering cisplatin’s anticancer efficacy [274]. Therefore, I3C can be used to decrease the nephrotoxicity induced by cisplatin and the combination of these two compounds improves the anticancer activity of each other.

Doxorubicin (DOX) is a well-known anticancer agent that has been reported to induce cardiac toxicity in human patients through enhancing inflammation and oxidative stress [275]. I3C treatment significantly reduced DOX-induced cardiotoxicity through inhibition of inflammation and oxidative stress (activation of NRF1/HO-1/NQO1 pathway) [150] in mice cardiac cells and bone marrow. Moreover, a combination of DOX and I3C treatment showed better anticancer activity than their individual treatment [152]. Cyclophosphamide is on the list of essential medicines by the World Health Organization (WHO) to treat several cancers. However, it causes chromosomal aberrations in healthy cells [276,277]. I3C inhibited cyclophosphamide-induced chromosomal aberrations, indicating that it can be co-administered with cyclophosphamide to mitigate the associated abnormal effects [278,279]. In another study, cyclophosphamide-induced developmental changes such as tail malformations and decreased fetal limbs in mice were reduced with I3C treatment [280]. Hence, these findings suggest that I3C could be used to treat cyclophosphamide-induced genotoxicity. Bleomycin is used to treat uterus, cervix, head, neck, and testicular cancers [281]. However, bleomycin induces liver fibrosis (BIP) through elevating oxidative damage and inflammatory cytokines and promotes the collagen deposition and accumulation of extracellular matrix [282,283]. In the model of BIP, I3C improved total antioxidant capacity (T-AOC) in serum and decreased TGF-β, Smad, α-SMA, and collagen 1 mRNA expression, pulmonary index, and hydroxyproline content, suggesting that I3C can alleviate bleomycin-induced pulmonary fibrosis in mice by inhibiting the TGF- β /Smad signaling pathway [284]. Further, it has been identified that I3C ameliorated the N-methyl N-nitro-N-nitrosoguanidine-induced single-strand DNA breaks in lung CHV-79 cells (fibroblasts) in a dose- and time-dependent manner [285]. Cumulatively, I3C has been reported to alleviate these side effects without altering the anticancer effects of cisplatin, methotrexate, doxorubicin, cyclophosphamide, and bleomycin. Hence, I3C can be co-administered with these anticancer agents to combat their toxicities.

**Figure 3:**
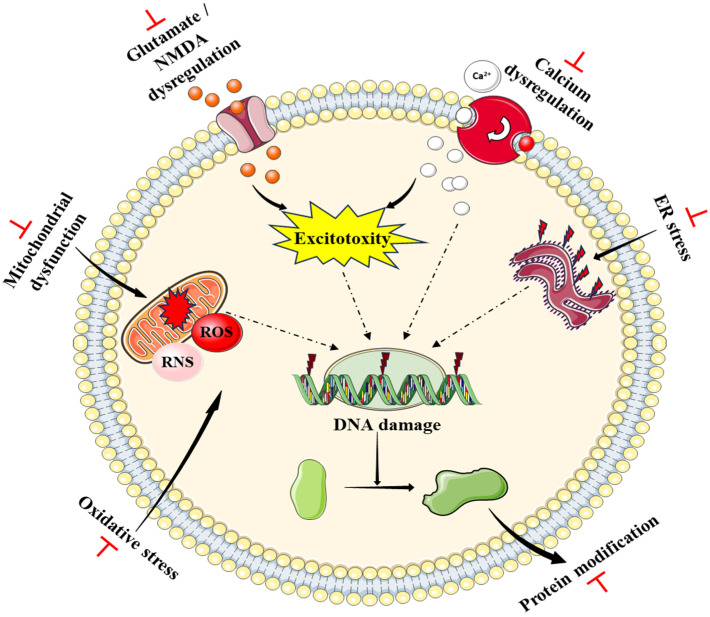
A schematic representation of multiple cellular events that are modified with I3C and DIM in the pathogenesis of various diseases. I3C and DIM mitigated mitochondrial dysfunction oxidative stress, glutamate (excitotoxicity) and calcium imbalances, ER stress, protein modifications, and DNA damage, thereby offering protection against various diseases, including cardiovascular, metabolic, neurological, etc.

Glucocorticoids (Dexamethasone) induce apoptosis in osteoblasts which further progresses to osteoporosis, bone loss, and oxidative stress [286]. I3C (5, 10, 20 μM for 2 h treatment) reduced the dexamethasone (1 μM treatment for 24 h)-induced cytotoxicity and cell death in MC3T3-E1 cells by decreasing caspase 9, 8, 3, and PARP in a sub-G1 cell population and upregulated antioxidant mediators like NRF2, HO1, and NQO1, indicating that I3Cs’ antioxidant and antiapoptotic actions prevented GC-induced osteoporosis [287]. Like its parent compound I3C, DIM enhanced bone formation as evidenced by osteogenic differentiation of MC3T3-E1 cells in vitro and enhanced the expression of various bone formation markers such as upregulation of BRCA1-Associated Protein 1 (BAP1), inositol 1,4,5-trisphosphate Receptor (IP3R), and storage operation calcium entry (SOCE)-related protein Recombinant Stromal Interaction Molecule 1 (STIM1) in osteoblasts. These findings suggest that the activation of the BAP1/IP3R/SOCE signaling pathway by DIM shows its osteogenic properties [288]. Clonidine is a well-known inducer of depression-like symptoms in rats such as a decrease in locomotor activity, self-interest, and emotionality as well as reduced exploratory motivation [289]. I3C increased the antioxidant enzymes (GSH, SOD) and decreased the proinflammatory markers and apoptotic factors after clonidine treatment in rats. I3C decreased noradrenaline and serotonin, indicating that I3C could be an antidepressant [132]. Ramakrishna et al. found that I3C treatment significantly mitigated isoproterenol-induced myocardial infarction through inhibiting oxidative stress and inflammation [169]. Cumulatively, these findings suggest that I3C mitigated the toxicities induced by dexamethasone, isoproterenol, cisplatin, clonidine, doxorubicin, cyclophosphamide, and bleomycin without altering their efficacies. Therefore, while using these drugs for their respective treatments, co-administration of I3C would be a promising approach to combat their toxicities. The various cellular protective mechanisms of I3C and DIM are depicted in Figure 3.

### 3.11. Reproductive Diseases

**Figure 4:**
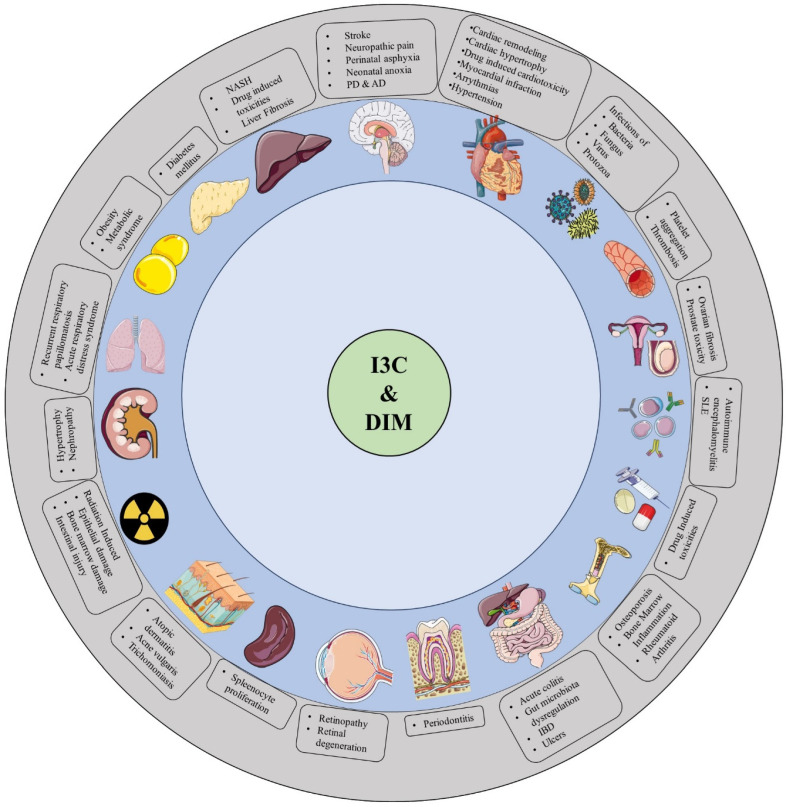
The protective responses of I3C and DIM on various diseases. I3C and DIM exhibited protective responses against neurological, cardiovascular, infectious, reproductive, respiratory, bone, dental, gastrointestinal, eye, autoimmune, spleen, skin, kidney, metabolic, pancreatic, and liver diseases, as well as drug-, chemical-, and radiation-induced toxicities.

Ovarian aging because of ovarian fibrosis, apoptosis, and oxidative damage leads to infertility and birth defects [290]. I3C (20 mg/kg) administration to old-age ICR mice (8 months) showed alleviation of ovarian damage due to aging by inhibiting oxidative stress via activation of the NRF2/HO-1 pathway. Further I3C also reduces the ovarian fibrosis and apoptosis caused by aging [291], indicating that I3C impedes ovary damage caused by aging. Polycarbonate plastics produce bisphenol A (BPA), which can induce reproductive toxicity and, in particular, prostate and mammary gland toxicity [292]. BPA (25 μg BPA/kg body weight) induction exhibited various prostate structural alterations such as increased inflammatory cells in the interstitial space and intraluminal papillary projections, and epithelial hyperplasia and acinar cells presented heterochromatic irregular nuclei, vacuolated mitochondria, and degenerated microvilli in male offspring, which were reversed by I3C treatment (2 g/kg in chow) [293,294]. Further, BPA reduced stromal compartments, and the area occupied by collagen in the prostate was reduced with the I3C treatment [295]. Similarly, in female offspring of Sprague-Dawley rats (post-natal day 21), I3C inhibited the BPA-induced increase in the number of terminal end buds, terminal ducts, and branching of mammary glands [296]. Hence, these findings suggest that I3C protects the male and female reproductive organs that are affected due to intoxication with BPA. Further research needs to investigate the protective and toxic effects of I3C and DIM on reproductive organs. The protective responses of I3C and DIM against various diseases are illustrated in Figure 4.

### 3.12. I3C- and DIM-Induced Toxicities

Although a vast body of literature suggests the beneficial effects of I3C and DIM in managing various diseases, their side effects or adverse events have not been yet documented in a cumulative report. Therefore, in this work, we have also summarized the reports of their toxic effects. Sub-chronic studies on F344 rats treated with I3C (≥150 mg/kg/oral) showed dose-related dilation of lymphangiectasia of the duodenum, jejunum, and mesenteric lymph nodes and extracellular lipid accumulation within the villar lamina propria, lacteals, and villar macrophages [297]. However, the protective mechanisms of I3C reported above are less than 150 mg/kg dose. Therefore, future studies are needed to understand the sub-chronic toxicity studies of I3C with a dose of less than 150 mg. Supplementation of I3C with high-fat diets resulted in a significant reduction in vitamin A concentration in blood plasma by 12% and in the liver by 37% [31]. Therefore, while I3C is recommended for reducing the fat content in the body, vitamin A levels must be taken into consideration and monitored accordingly.

It is well known that human pregnane X receptor (hPXR) activation regulates the expression of cytochrome P450 3A4 (CYP3A4) and multidrug resistance protein 1 (MDR1), which plays a crucial role in adverse drug interactions and drug metabolism. DIM significantly activates the hPXR leading to acceleration of the expression of CYP3A4 and MDR1. Therefore, it is recommended that simultaneous use of DIM with other medications that are metabolized rapidly by CYP3A4 and MDR1 [298] be avoided. I3C (250, 500, and 750 mg/kg) supplementation to CD mice through diet has dose-dependently decreased serum testosterone levels when compared to control animals [299]. However, the selected dose for this study was relatively high and none of these doses have been studied by researchers for reporting biological activities. So, while using I3C in males, testosterone levels must be considered. In a study to test the potential of I3C on the induction of hepatocyte hypertrophy and cytochrome P450 (CYP) activity, I3C treatment for 28 days with the dose of 50 mg/kg significantly increased the liver weights as well as CYP 1A1/2, 2B1/2, 2C9, 2D6, 2E1, 3A4, and 19A in male rats compared to female rats. However, DIM treatment (75 mg/kg, oral) for 28 days showed an insignificant increase in liver weights and CYP levels and these effects were normalized after withdrawal of DIM. Moreover, I3C withdrawal also reduced hepatocyte hypertrophy and CYP enzyme levels. Therefore, it has been suggested that while using I3C, changes in the liver function and production of CYP enzymes must be monitored closely [300].

Herz et al. reported that I3C and DIM inhibited telomerase in normal human immune cells (peripheral blood mononuclear cells (PBMCs)). Similarly, I3C and DIM inhibited telomerase activity in leukemia cells [301]. While these findings are promising for leukemia treatment, using I3C and DIM in non-cancerous diseases could pose a risk. Specifically, inhibiting telomerase in healthy PBMCs might affect immune function. However, these reported findings are only from in vitro studies. The identification of similar effects in vivo should be carried out in future studies. Six-to-seven-day-old C57BL/6 mice, following DIM oral treatment (20, 60, and 100 mg/kg) for three days, showed immunotoxin responses. DIM (100 mg/kg) decreased various immune cells like F4/80+, CD11C+, CD19+, and CD3+ and induced splenic white pulp atrophy, increased immune cell apoptosis, decreased the expression of TLRs in the spleen and small intestine, and upregulated caspase-3 in the neonatal mouse. However, whilst these effects were seen with DIM 20 and 60 mg/kg, they are statistically insignificant. Thus, this study confirms that DIM acts as an immunotoxin in newborn mouse models and may provide valuable clues for the development of a safe supplement, especially one designed for mouse and human infants [302]. However, future studies need to determine whether similar effects will be shown in adult mice. Despite numerous beneficial effects, I3C and DIM also exhibited a few side effects. Moreover, most of these toxic effects of I3C and DIM occurred at their higher doses, but not at therapeutic doses. Therefore, while using these compounds, these side effects must be considered to avoid the undesired outcomes. The toxic effects of I3C and DIM are depicted in Table 2.

## 4. Challenges and Opportunities

I3C and DIM exhibited numerous protective responses across all body organs through their antioxidant, anti-inflammatory, antiapoptotic, immunomodulatory, and xenobiotic mechanisms. Nevertheless, there are several disadvantages associated with I3C, such as low half-life, due to rapid kidney clearance and rapid inactivation by metabolic enzymes, instability in gastric pH, low molecular weight, and low water solubility. To overcome some of these limitations of I3C, several novel oral formulations (microparticles and hydrogel-containing nanocapsules) have been prepared that have led to improved bioavailability and therapeutic effects [120,121,122,188,241,303,304]. However, despite this, the maximum therapeutic outcomes of I3C and DIM have not been achieved. Therefore, future studies must design and develop novel formulations that could achieve the maximum therapeutic utility of these compounds. Currently, I3C and DIM are available as over-the-counter nutraceuticals to support healthy estrogen metabolism, detoxification, immune function, antioxidant effects, etc. Many of the health benefits discussed above have been studied in preclinical stages using animals or in vitro studies. Few clinical trials have reported the anticancer potential of I3C and DIM [304,305,306,307,308,309]. While these clinical trials have focused on their anticancer properties, they have reported that I3C and DIM may serve as promising phytochemicals derived from cruciferous vegetables with potential for drug development. Indeed, there have been no clinical trials studying I3C and DIM against the above-mentioned diseases, except for cancer. Therefore, future studies must investigate the clinical potential of I3C and DIM. Moreover, preclinically, the antioxidant and immunomodulatory roles of I3C and DIM have been clearly defined in various diseases. However, rigorous clinical trials evaluating the optimal dosages and duration, bioavailability, long-term safety, and therapeutic benefits of these compounds must be assessed for their therapeutic viability.

Overall, I3C and DIM are reported to have various health benefits against cardiovascular, neurological, reproductive, metabolic, bone, infectious, respiratory, liver, and immune diseases, as well as drug-induced toxicities. There are still abundant avenues available for I3C and DIM to become druggable candidates with future investigations.

## Figures and Tables

**Table 1:** Antimicrobial effects of I3C and DIM and their derivatives.

S. No.	Compound	Activity	Target Species	Mechanism of Action
1	DIM	Antiprotozoal	*L. donovani*	Inhibits mitochondrial F0F1-ATP synthase and induces apoptosis [183]. Inhibits DNA topoisomerase I non-competitively, causing parasite death [183]. Causes mutations in *Leishmania* DNA topoisomerase IB (LdTOP1LS), leading to resistance [185].
2	DIM Analogs	Antiprotozoal	*L. donovani*	Overcomes DIM resistance and retains antileishmanial activity [186].
3	I3C	Antiprotozoal	*Trichomonas vaginalis*	Vaginal hydrogel with I3C-loaded nanocapsules reduced parasite viability [188].
4	I3C	Antifungal	*Candida albicans*	Binds and disrupts DNA [192]. Induces cell cycle arrest at G2/M phase and disrupts membrane structure [194].
5	I3C	Antibacterial	*Gram-positive bacteria*	More effective in Gram-positive than Gram-negative bacteria due to lack of LPS later and synergistic with ampicillin [197].
6	I3C	Antibacterial	*Clostridium difficile*	Reduced CDI severity and mortality, improved gut microbiota composition, and enhanced cecal Tregs, γδ T cells, ILC3s, and neutrophilic response without increasing inflammatory response and bacterial translocation [199].
7	I3C derivatives	Antibacterial	*Staphylococcus aureus*	Ethyl acetate extracts of I3C-exposed *Daldinia eschscholzii* fungal produced unique indole alkaloids, such as dalesindoloids A and B which inhibit bacterial growth [200].
8	I3C derivatives	Antibacterial	*C. difficile*, *C.perfringens*, *B. fragilis*, *S.pyogenes*, and *Veillonella species*	I3C-exposed *Daldinia eschscholzii* produced PIHs (indolchromins A and B) to inhibit bacterial growth [201].
9	I3C	Antibacterial	*E. coli*	Induces ROS and DNA damage thereby causing apoptosis-like death [203].
10	I3C and DIM	Antibacterial	*C. acnes*, *S. aureus*, *C. albicans*	I3C and DIM inhibited bacterial growth and biofilm formation. DIM inhibited hyaluronate lyase, virulence-related genes, and lipase [205].
11	DIM	Antibacterial	*P. aeruginosa*, *A. baumannii*	DIM inhibits biofilm formation (70–94% inhibition with tobramycin) [206].
12	I3C	Antibacterial	MDR Gram-negative bacteria (*A. baumannii*, *K.pneumoniae*, *P. aeruginosa*, *E. coli*)	I3C showed synergistic bactericidal activity with CCCP and EDTA [208].
13	I3C	Antiviral	*Human papillomavirus*	Alters estrogen metabolism and reduces papilloma growth [9].
14	I3C	Antiviral	*Herpes simplex virus (HSV1*, and *HSV2)*	Promotes p53, p21, and cyclin D3. Inhibits DNA synthesis at G1/S phase [211].
15	I3C	Antiviral	*SARS-CoV-2*	Downregulates IFN-related genes (*IFIT1*, *TRIM22*, and *MX2*). Inhibits viral replication [213].
16	I3C	Antiviral		Inhibits NEDD4 E3 ubiquitin protein ligase (NEDD4) and WWP1, thereby impeding viral egression [214].
17	DIM Derivatives	Antiviral		Inhibits SARS-CoV-2 main protease (Mpro), blocking viral transcription [215].

**Table 2:** The toxic effects of I3C and DIM.

Compound	Toxic Effect	Dose and Duration	Organ/System Affected	Findings and Notes	Species
I3C	Lymphangiectasia and extracellular lipid accumulation	≥150 mg/kg (oral); sub-chronic	Duodenum, jejunum, and mesenteric lymph nodes	Dose-related dilation of lymphatic vessels, lipid accumulation in villar lamina propria, lacteals, and villar macrophages	F344 rats [297]
	Reduction in vitamin A levels	High-fat diet with I3C	Blood plasma and liver	12% reduction in plasma vitamin A and 37% reduction in liver vitamin A	Wistar rats [31]
	Enzyme induction	50 mg/kg (oral) for 28 days	Liver	Increased hepatocyte hypertrophy and CYP1A1/2, 2B1/2, 2C9, 2D6, 2E1, 3A4, and 19A enzyme levels (more pronounced in males)	Male and female rats (CD^®^VAF) [300]
	Decrease in serum testosterone levels	250, 500, and 750 mg/kg (oral)	Endocrine (testosterone levels)	Dose-dependent reduction in serum testosterone	CD-1 mice [299]
DIM	Drug interaction via hPXR activation	In vitro study	Drug metabolism (CYP3A4, MDR1)	Increases CYP3A4 and MDR1 expression; caution is needed with medications metabolized by these enzymes	In vitro (human PXR activation) [298]
	Liver toxicity	75 mg/kg (oral) for 28 days	Liver	Insignificant liver weight increase and CYP enzyme activation; normalizes after withdrawal	Male rats and female rats (CD^®^VAF) [300]
	Immunotoxic effects in neonatal mice	20, 60, 100 mg/kg (oral) for 3 days	Immune system; spleen	White pulp atrophy; immune cell apoptosis; reduced TLR expression; increased caspase-3	C57BL/6 neonatal mice [302]
I3C and DIM	Telomerase inhibition	In vitro study	Peripheral blood mononuclear cells (PBMCs); leukemia cells	Telomerase inhibition in normal PBMCs and leukemia cells; possible immune function risks in non-cancer patients	In vitro (human PBMCs, leukemia cells) [301]

## Data Availability

All data generated or analyzed during this study are included in this published article.
